# Crop-associated differentiation of rhizosphere microbial communities and soil functional traits in saline-alkaline farmland

**DOI:** 10.3389/fmicb.2026.1952914

**Published:** 2026-10-15

**Authors:** Xiaoling Zhang, Yuhao Yuan, Yanpeng Shi, Siyuan Chen, Zhijun Gao, Qinghua Yang, Hanghang Hou, Baili Feng

**Affiliations:** 1 State Key Laboratory of Crop Stress Biology for Arid Areas, College of Agronomy, Northwest A&F University, Yangling, Shaanxi, China; 2 National Key Laboratory of Wheat and Maize Crop Science, College of Agronomy, Henan Agricultural University, Zhengzhou, Henan, China; 3 Shaanxi Land Engineering Construction Group Co., Ltd., Xi’an, Shaanxi, China; 4 Inner Mongolia Erdos Institute of Agriculture and Animal Husbandry, Crop Research Institute, Erdos, Inner Mongolia, China

**Keywords:** crop identity, multiple factor analysis, plant ion allocation, rhizosphere microbiome, saline-alkaline soil

## Abstract

**Introduction:**

Saline-alkaline soils constrain crop production, yet it remains unclear how different crops shape rhizosphere conditions, plant ion allocation and microbial communities when grown under the same field background.

**Methods:**

We compared eight crops under uniform saline-alkaline field and management conditions and integrated rhizosphere physicochemical properties, enzyme activities, plant nutrient and ion traits, and bacterial and fungal community characteristics using a multivariate framework.

**Results:**

Crop identity was associated with pronounced differences in nutrient status, salinity, moisture, enzyme activity and Na^+^ and K^+^ allocation, and explained 78.1% and 83.5% of bacterial and fungal community variation, respectively. Multiple factor analysis further separated the eight crops into two stable multivariate profiles, one characterized mainly by nutrient and enzyme traits and the other by higher salinity and greater contributions from Na^+^ allocation traits.

**Discussion:**

These findings demonstrate that crop-associated differentiation in saline-alkaline farmland reflects coordinated variation among soil conditions, plant ion regulation and microbial organization, providing an integrated basis for crop comparison and saline-alkaline soil management.

## Introduction

Salinized soils cover approximately 1.38 billion ha worldwide and the area affected by salinization continues to increase each year (FAO, 2024). Salinity restricts crop growth through osmotic stress, Na^+^ and Cl- toxicity, and deterioration of soil aggregation, aeration and water permeability (Ma et al., 2026; Wong et al., 2010; Zhao et al., 2020). Saline-alkaline stress can alter microbial N and P cycling, including changes in nitrification, denitrification and nutrient-related functional potential (Pang et al., 2024; Yang et al., 2025). These linked effects can reinforce declines in soil fertility and crop performance (Ondrasek and Rengel, 2021). Crop-based rehabilitation is attractive because it can combine productive land use with gradual soil improvement (Tarolli et al., 2024). Through root exudation, nutrient uptake and rhizosphere pH modification, crops can select microbial communities that influence nutrient availability and plant stress responses (Zhao et al., 2021; Zheng et al., 2024). These reciprocal plant–soil-microbe feedbacks may help explain why crops differ in their performance on saline-alkaline land (Walder et al., 2023; Zhao et al., 2021).

Saline-alkaline stress alters microbial community structure, function and nutrient cycling. It can inhibit genes involved in nitrogen (N) and phosphorus (P) metabolism, impair nitrification, promote denitrification and suppress P desorption, mineralization and solubilization (Pang et al., 2024; Yang et al., 2025). These changes can reduce soil N and P availability. Microbial responses to saline-alkaline stress involve shifts in community organization and functional potential, while root-exudate-mediated feedbacks can further modify soil-microbe-nutrient cycling (Chen et al., 2024; Zhao et al., 2021). Core microbial taxa and network organization can contribute to plant–soil resilience during ecosystem restoration (Du et al., 2025). In legumes such as alfalfa, plant functional traits and stress-responsive metabolites are linked to rhizosphere microbial interactions under saline-alkaline stress (Liu et al., 2023; Liu et al., 2024). Microbial extracellular polymers and fungal hyphae can also promote aggregation through physical entanglement and chemical bonding (Close et al., 2026; Feng et al., 2026). Crop identity, soil conditions and rhizosphere communities are therefore connected through reciprocal feedbacks.

Although recent work has begun to compare crop-specific rhizobacteriomes across several crops (Abdelrahman et al., 2025), it remains difficult to separate crop-specific patterns from features shared across saline rhizospheres. Existing single-crop studies have linked microbial community composition with multifunctionality or soil physicochemical properties (Wang et al., 2023; Jiang et al., 2025), but comparative evidence integrating soil, plant-ion and microbial community characteristics across diverse crops remains limited. A common-field comparison is therefore needed to identify crop-associated differences in soil conditions, microbial diversity, community composition and potential functional organization under the same soil and management background.

To address these gaps, we compared eight crops with contrasting botanical affiliations, root systems, nutrient-acquisition strategies and agricultural uses. The species were alfalfa (*Medicago sativa* L.; Fabaceae), broomcorn millet (*Panicum miliaceum* L.; Poaceae), Jerusalem artichoke (*Helianthus tuberosus* L.; Asteraceae), oat (*Avena sativa* L.; Poaceae), quinoa (*Chenopodium quinoa* Willd; Amaranthaceae), rapeseed (*Brassica napus* L.; Brassicaceae), sugar beet (*Beta vulgaris* L.; Amaranthaceae) and sunflower (*Helianthus annuus* L.; Asteraceae). Together, these species represent legumes, cereals, pseudocereals, oilseeds, root crops and tuber crops with contrasting salt tolerance and rhizosphere traits. We combined soil physicochemical measurements with bacterial 16S rRNA gene and fungal ITS amplicon sequencing. The objectives were to compare crop-associated differences in rhizosphere soil properties, characterize bacterial and fungal diversity, composition and co-occurrence organization, and identify soil variables associated with microbial community differentiation. This common-field comparison was designed to generate evidence relevant to crop selection and future rotation studies in saline-alkaline agriculture.

## Materials and methods

### Plant materials and seed treatment

Eight crop species were used. Seven species were established from seed, whereas Jerusalem artichoke was established from tubers. The seed-propagated species were alfalfa (*Medicago sativa* L., cv. Zhongmu No. 5; AF), broomcorn millet (*Panicum miliaceum* L., cv. Yimi No. 11; BM), oat (*Avena sativa* L., cv. Mengyan No. 3; OT), quinoa (*Chenopodium quinoa* Willd., cv. Mengli No. 1; QN), rapeseed (*Brassica napus* L., cv. Huayouza No. 62; RS), sugar beet (*Beta vulgaris* L., cv. Neitiankang 201; SB) and sunflower (*Helianthus annuus* L., cv. Sanrui 361; SF). Uniform, healthy Jerusalem artichoke tubers (*Helianthus tuberosus* L., cv. Mengyu No. 4; JA) were used. These cultivars are grown in saline-alkaline farmland in Ordos City, Inner Mongolia, and were supplied by the Ordos Academy of Agriculture and Animal Husbandry Sciences. Alfalfa, broomcorn millet, quinoa and rapeseed were treated as small-seeded crops for the sowing protocol, whereas oat, sugar beet and sunflower were treated as large-seeded crops. Pregermination tests for the seed-propagated crops indicated germination of at least 92% and purity of at least 98%. Shrivelled, damaged or contaminated seeds were removed manually. Seeds were immersed in 75% (v/v) ethanol for 30 s, gently shaken in 1% (v/v) sodium hypochlorite at 110 rpm for 5 min, rinsed six times with sterile deionized water and air-dried in a laminar-flow cabinet at 23 °C ± 2 °C until they returned to their initial moisture content of 12%–14%. Uniform Jerusalem artichoke tubers without visible damage or disease symptoms were selected separately. After surface sterilization, the tubers were cut with a sterilized scalpel into uniform tuber pieces with a fresh weight of 30–35 g. Each tuber piece retained two to three intact and vigorous bud eyes to ensure consistent germination potential. Damaged or diseased tubers were discarded before planting.

### Study site and soil characteristics

The field experiment was conducted at a saline-alkaline experimental station on the northern Loess Plateau, China (40°47′N, 110°24′E; 1,026 m above sea level). The region has a temperate continental semi-arid climate. The mean annual temperature ranges from 5.3 °C to 8.7 °C, mean annual precipitation is 265.34 mm and occurs mainly from July to September, and annual evaporation ranges from 1,800 to 2,200 mm. Before the experiment, composite topsoil samples (0–20 cm) were collected from five evenly distributed locations using an S-shaped sampling pattern. The samples were homogenized before baseline physicochemical analysis. The sandy loam contained 68.5% sand, 22.3% silt and 9.2% clay. Initial pH was 8.82, and the electrical conductivity of the 1:5 soil-to-water extract was 3.21 dS m^−1^. Total salt content was recalculated as EC_1:5_ × 5 × 0.064, giving 1.03%. Total P, K and N were 0.45, 21.94 and 0.35 g kg^−1^, respectively. Based on FAO diagnostic standards for salt-affected soils, the soil was classified as salt-affected alkaline soil with a strongly alkaline pH (FAO, 2024).

### Experimental design and field management

The experimental field had been uniformly planted with maize in the preceding growing season, which was harvested in October of the previous year. Aboveground crop residues were removed after harvest, and the entire field subsequently received the same pre-sowing tillage and management before establishment of the present experiment. Thus, all experimental plots shared the same preceding-crop history before the eight crop treatments were imposed. The experimental field covered 1,650 m^2^ (55 m × 30 m). Before sowing, deep tillage, rotary tillage and harrowing were used to homogenize the topsoil. The site was level and had unobstructed drainage. The experiment used a randomized complete block design with eight crop treatments and three biological replicates per treatment, giving 24 plots. Treatments were randomly assigned within each block. Each plot measured 6 m × 5 m (30 m^2^). Adjacent plots were separated by 1-m bare buffers, blocks by 2-m isolation strips, and the experimental area by 4-m protective rows planted with millet. Conventional crop-specific spacing was used (Table 1). Soil cover and compaction were standardized after planting. Sunflower, sugar beet and Jerusalem artichoke were planted on 5 May, and the remaining crops were sown on 25 May. The same field-management practices were applied across all plots throughout the growing season.

**Table 1:** Planting and sampling information for the eight crops.

Crop name	Row spacing (cm)	Plant spacing (cm)	Sowing depth (cm)	Sowing date	Sampling date	Phenological stage
Alfalfa	30	5	3	25 May 2025	6 Aug 2025	Full flowering stage
Broomcorn millet	33	5	3	25 May 2025	6 Aug 2025	Full flowering stage
Quinoa	30	5	3	25 May 2025	21 Jul 2025	Full flowering stage
Rapeseed	30	5	2	25 May 2025	21 Jul 2025	Full flowering stage
Sugar beet	40	20	3	5 May 2025	21 Jul 2025	Rapid taproot enlargement stage
Sunflower	40	50	3	5 May 2025	21 Jul 2025	Full flowering stage
Jerusalem artichoke	50	40	10	5 May 2025	21 Jul 2025	Full flowering stage
Oat	15	5	5	25 May 2025	21 Jul 2025	Full flowering stage

Before sowing, all plots received 160 kg ha^−1^ urea (46% N; 73.6 kg N ha^−1^), 320 kg ha^−1^ superphosphate (providing 16.8 kg P ha^−1^) and 130 kg ha^−1^ potassium sulfate (providing 54.0 kg K ha^−1^). Urea was applied as a single basal dose. Fertilizers were incorporated evenly into the topsoil by plowing. Drip irrigation was applied at the seedling and flowering stages of each crop using a quantified water supply. Plots were weeded manually every 2 weeks, and weed residues were removed. No synthetic pesticides were applied during cultivation.

Rhizosphere sampling was scheduled according to crop phenology. Quinoa, rapeseed, sugar beet, sunflower, Jerusalem artichoke and oat were sampled on 21 July 2025, whereas alfalfa and broomcorn millet were sampled on 6 August 2025. Quinoa, rapeseed, sunflower, Jerusalem artichoke, oat, alfalfa and broomcorn millet were sampled at the full flowering stage, whereas sugar beet was sampled at the rapid taproot enlargement stage. The two sampling dates were separated by 16 d. Phenology-based sampling was adopted to reduce developmental-stage heterogeneity among crops, because root-derived inputs, nutrient demand and rhizosphere microbial processes can vary markedly with plant developmental stage. No irrigation or other major field-management operation was conducted between the two sampling dates. Exact sowing dates, sampling dates and phenological stages are provided in Table 1. Whole plants were carefully uprooted, and loose bulk soil was removed by gentle shaking. Soil that remained tightly attached to the roots was collected as rhizosphere soil. Five plants from the centre of each plot were pooled to form one biological replicate. Approximately 500 g of pooled rhizosphere soil was collected per plot in a sterile sampling bag. Samples were homogenized and divided into aliquots for molecular, enzyme and physicochemical analyses. Molecular aliquots were transferred to sterile tubes, transported on ice and stored temporarily at −80 °C. The remaining soil was processed fresh or air-dried according to the analytical method. Three independent biological replicates were collected per crop treatment.

### Determination of soil physicochemical properties and enzyme activities

Soil physicochemical properties were measured in air-dried soil unless otherwise stated. Soil pH was measured with a PHS-3C meter (INESA Scientific Instrument Co., Shanghai, China) in a 1:2 soil-to-water suspension. Total N was measured with a FOSS 8400 automated Kjeldahl analyser. Organic C was determined by potassium dichromate titration. Total P and total K were quantified by inductively coupled plasma analysis after microwave digestion. Ammonium N was measured by the indophenol-blue method, nitrate N by dual-wavelength ultraviolet spectrophotometry, and alkali-hydrolysable N by alkaline-hydrolysis diffusion. Water-soluble Na was measured by flame photometry. Total soluble salts were estimated from the electrical conductivity of a 1:5 soil-to-water extract following Pang et al. (2010). The suspension was shaken for 5 min, and total salts were calculated as TS (%) = EC_1:5_ × 5 × 0.064. Gravimetric moisture content was determined by drying 10 g of fresh soil at 105 °C to constant mass. Moisture content was calculated as (M − m)/m × 100%, where M and m are the initial and dry masses, respectively. Enzyme activities were measured in fresh soil. Urease activity was determined by the phenol-sodium hypochlorite method, and dehydrogenase activity was measured by reduction of 2,3,5-triphenyltetrazolium chloride. Enzyme activities were measured independently in the three biological replicates for each crop treatment. Biological replicates, rather than repeated instrument readings, were used for statistical inference.

### Determination of plant nutrient and ion traits

For plant nutrient and ion analyses, plant samples collected from each plot were separated into aboveground tissues and roots. Roots were carefully washed to remove adhering soil and subsequently rinsed with deionized water. The aboveground and root samples were first heated at 105 °C for 30 min to terminate enzymatic activity and then oven-dried at 85 °C to constant weight. The dried samples were ground into a homogeneous fine powder and stored in sealed containers until chemical analysis.

Ground plant material was digested using an H₂SO₄–H₂O₂ digestion procedure. Total nitrogen (TN) was determined by the Kjeldahl method using a FOSS 8400 automated Kjeldahl analyser. Total phosphorus (TP) and total potassium (TK) were quantified by inductively coupled plasma analysis, whereas Na concentration was determined by flame photometry. Aboveground tissues and roots were analysed separately for each of the three biological replicates per crop.

The Na^+^/K^+^ ratio was calculated separately for aboveground tissues and roots as

$$\mathrm{Na}/K=\frac{\mathrm{Na}}{\mathrm{TK}}$$

The Na and K translocation factors were calculated from the concentration ratios between aboveground and root tissues as

$${\mathrm{TF}}_{\mathrm{Na}}=\frac{{\mathrm{Na}}_{\text{shoot}}}{{\mathrm{Na}}_{\text{root}}},{\mathrm{TF}}_{K}=\frac{{\mathrm{TK}}_{\text{shoot}}}{{\mathrm{TK}}_{\text{root}}}$$

K–Na translocation selectivity was calculated as

$$S_{K,\mathrm{Na}}=\frac{{\left(\mathrm{TK}/\mathrm{Na}\right)}_{\text{shoot}}}{{\left(\mathrm{TK}/\mathrm{Na}\right)}_{\text{root}}}$$

### DNA extraction, sequencing, and bioinformatic analysis

A total of 24 rhizosphere soil samples were collected from alfalfa, broomcorn millet, Jerusalem artichoke, oat, quinoa, rapeseed, sugar beet and sunflower. For each biological replicate, genomic DNA was extracted from 0.25 g of homogenized fresh rhizosphere soil using the E. Z. N. A. Soil DNA Kit (Omega Bio-tek, Inc., USA) according to the manufacturer’s instructions. DNA concentration and purity were assessed using a NanoDrop 2000 spectrophotometer (Thermo Scientific, USA), and extracted DNA was stored at −20 °C. The V3-V4 region of the bacterial 16S rRNA gene was amplified with primers 338F (5′-ACTCCTACGGGAGGCAGCAG-3′) and 806R (5′-GGACTACNNGGGTATCTAAT-3′). The fungal ITS1 region was amplified with primers ITS1F (5′-CTTGGTCATTTAGAGGAAGTAA-3′) and ITS2 (5′-GCTGCGTTCTTCATCGATGC-3′). An eight-base barcode was added to the 5′ end of each primer for every sample (Allwegene, Beijing, China). Bacterial and fungal PCRs were performed on an ABI 9700 instrument in 25-μL reactions containing 12.5 μL of 2 × Taq PCR Master Mix, 3 μL of BSA (2 ng μL^−1^), 1 μL of each primer (5 μM), 2 μL of template DNA and 5.5 μL of double-distilled water. Cycling began with 5 min at 95 °C, followed by 28 cycles of 45 s at 95 °C, 50 s at 55 °C and 45 s at 72 °C, with a final extension of 10 min at 72 °C. Products were purified with an Agencourt AMPure XP Kit (Beckman Coulter, USA). Libraries were prepared with the NEBNext Ultra II DNA Library Prep Kit (New England Biolabs, USA). Library quality was assessed using a NanoDrop 2000, an Agilent 2100 Bioanalyser and an ABI StepOnePlus Real-Time PCR System. Paired-end 300-bp sequencing was performed on an Illumina MiSeq PE300 platform using a MiSeq Reagent Kit v3 (600 cycles) at Beijing Allwegene Technology Co., Ltd. Image analysis, base calling and error estimation used Illumina Analysis Pipeline version 2.6. Reads were demultiplexed by barcode. PEAR v0.9.6 (Zhang et al., 2014) was used to filter and merge paired reads. Reads containing ambiguous bases were removed, and regions with quality scores of 20 or lower were trimmed. Merging required at least 10 bp of overlap and used a *p* value threshold of 0.0001. VSEARCH v2.7.1 (Rognes et al., 2016) removed bacterial sequences shorter than 230 bp and fungal sequences shorter than 120 bp. Chimeras were identified with UCHIME (Edgar et al., 2011) against the Gold database for bacteria and the UNITE database for fungi. Qualified sequences were clustered into operational taxonomic units (OTUs) at 97% similarity using UPARSE in VSEARCH (Edgar, 2013; Rognes et al., 2016). Representative bacterial and fungal sequences were classified with BLAST (Ye et al., 2006) against SILVA 138 and UNITE 8.2, respectively. An E value threshold of 1 × 10^−5^ was used (Abarenkov et al., 2010; Pruesse et al., 2007). Mitochondrial and chloroplast reads were removed before abundance, taxonomy, metadata and phylogenetic-tree objects were combined for analysis.

### Statistical analysis

Statistical analyses were performed using R version 4.3.1, Origin 2022 and SPSS version 26.0 (IBM Corp., Armonk, NY, USA). Normality and homogeneity of variance were evaluated with Shapiro–Wilk and Levene tests, respectively. Soil properties, enzyme activities, and plant nutrient and ion traits were compared among crops by one-way analysis of variance followed by Tukey’s honestly significant difference test when model assumptions were met. Differences in bacterial and fungal alpha-diversity indices among crops were evaluated using the Kruskal–Wallis test. To account for multiple testing across the six alpha-diversity indices (Chao1 richness, Shannon diversity and Faith’s phylogenetic diversity for bacteria and fungi), the resulting global *p* values were adjusted using the Benjamini–Hochberg false discovery rate (FDR) procedure. For indices showing a significant overall crop effect (FDR-adjusted *p* < 0.05), pairwise comparisons among crops were performed using Dunn’s *post hoc* test, with Benjamini–Hochberg correction applied to the pairwise *p* values within each index. Different lowercase letters indicate significant pairwise differences at adjusted *p* < 0.05. QIIME v1.8.0 (Caporaso et al., 2010) was used to generate rarefaction curves and calculate OTU-based richness and diversity indices, which were plotted in Origin 2022. Beta-diversity differences were tested by permutational multivariate analysis of variance based on Bray–Curtis dissimilarities with 999 permutations. Redundancy analysis (RDA) was used to evaluate associations between soil variables and microbial community composition. Community matrices were Hellinger-transformed before RDA. Model and predictor significance was assessed using 999 permutations. Environment-responsive OTUs were further identified using permutation-based vector fitting (envfit; 999 permutations), ranked according to their goodness of fit to the ordination, and the top 10 bacterial and fungal OTUs were retained for presentation. *p* values from the envfit analysis were adjusted using the Benjamini–Hochberg false discovery rate procedure. Linear discriminant analysis effect size identified differentially represented taxa using an LDA score greater than 3.5 and *p* < 0.05 (Segata et al., 2011). Pairwise relationships among rhizosphere environmental variables were assessed using Pearson correlation coefficients. Associations between microbial community composition and individual environmental variables were evaluated separately for bacteria and fungi using Mantel tests. Bray–Curtis dissimilarities were calculated for microbial community matrices, and Euclidean distances were calculated for environmental variables. Statistical significance of Mantel correlations was evaluated with 999 permutations. The resulting Pearson correlations and Mantel statistics were visualized using the ggcor package in R. The plot-level biological replicate was the statistical unit for soil properties, enzyme activities, plant nutrient and ion traits, and alpha diversity (*n* = 3 per crop). Repeated instrument readings, when present, were averaged before statistical analysis. Unless otherwise stated, *p* < 0.05 was considered statistically significant.

Taxonomic relative-abundance bar plots were generated in R. PICRUSt2 was used as an exploratory, hypothesis-generating analysis to infer potential bacterial gene-family and pathway profiles from the 16S rRNA amplicon data. Predicted features were annotated against Kyoto Encyclopedia of Genes and Genomes (KEGG) orthologs and pathways, and STAMP v2.1.3 was used for visualization. Because PICRUSt2 predictions are inferred from marker-gene data and reference genomes rather than directly measured by metagenomics, metatranscriptomics or metaproteomics, these results were interpreted only as putative functional potential and not as evidence of pathway expression or activity. Microbial co-occurrence networks were constructed by integrating bacterial and fungal OTUs. OTUs with a mean relative abundance below 0.01% or occurring in fewer than 50% of the samples were removed before network construction. Pairwise associations among the retained OTUs were evaluated using Spearman rank correlations, and *p* values were adjusted using the Benjamini–Hochberg procedure. Only associations with |r| ≥ 0.8 and adjusted *p* ≤ 0.05 were retained to construct a weighted, undirected reference network, and isolated nodes were removed. Crop-specific subnetworks were subsequently extracted from the reference network by retaining OTUs detected in the corresponding crop samples while preserving their associations in the reference network. Network modules were identified using the fast-greedy modularity optimization algorithm implemented in the igraph package. Network topology was characterized using node and edge numbers, positive and negative associations, average degree, average path length, network diameter, network density, clustering coefficient, centralization indices, modularity, information centrality and network complexity. Node topological roles were further evaluated using within-module connectivity (Zi) and among-module connectivity (Pi). Nodes with Zi > 2.5 and Pi < 0.62 were classified as module hubs, those with Zi < 2.5 and Pi > 0.62 as connectors, those with Zi > 2.5 and Pi > 0.62 as network hubs, and the remaining nodes as peripherals. Network visualization was performed using Gephi.

### Multiple factor analysis and cluster stability assessment

A total of 44 variables were organized into six quantitative blocks for MFA: soil nutrients (7 variables), soil salinity and moisture (4), enzyme activities (2), plant nutrient and ion traits (12), microbial diversity and community structure (10), and network and inferred functional characteristics (9).

MFA was performed using the FactoMineR package in R (Lê et al., 2008), with all blocks specified as standardized quantitative groups (type = “s”). Missing values, when present, were replaced by the median of the corresponding variable. Within the MFA framework, quantitative variables were standardized and each block was weighted by the inverse of its first singular value obtained from a separate principal component analysis, thereby balancing the influence of blocks differing in variable number or overall variance. Group and variable contributions to the MFA dimensions were subsequently extracted.

The number of MFA dimensions retained for clustering was defined as the smallest number between two and five that accounted for at least 70% of cumulative explained variance. Crop centroids were calculated by averaging the retained MFA coordinates of the three biological replicates for each crop. Euclidean distances among crop centroids were subjected to Ward.D2 hierarchical clustering. Candidate cluster solutions from k = 2 to 6 were compared using average silhouette width, and the solution with the highest value was retained. Cluster stability was assessed using 1,000 bootstrap iterations (seed = 123). In each iteration, biological replicates were resampled with replacement within each crop, followed by repetition of the MFA and Ward.D2 clustering procedure. Pairwise co-clustering frequencies were calculated as the proportion of bootstrap iterations in which two crops were assigned to the same cluster. Silhouette analysis and graphical visualization were conducted using the cluster, factoextra, ggplot2 and pheatmap packages.

## Results

### Crop species generated distinct rhizosphere soil nutrient, salinity and enzyme profiles

Rhizosphere physicochemical properties differed markedly among the eight crops (Figure 1; Supplementary Figure S1; Table 2). Alfalfa had the highest TN, OC and TP contents (Figure 1A; Supplementary Figures S1a,b), with values of 0.64 ± 0.02, 9.98 ± 0.24 and 0.82 ± 0.02, respectively, whereas rapeseed had the highest TK content (21.26 ± 0.51; Supplementary Figure S1c). Nitrogen forms also varied substantially among crops: rapeseed showed the highest NH₄^+^–N content (6.73 ± 0.07), quinoa the highest NO₃^−^–N content (22.17 ± 0.13), and oat the highest available N content (56.49 ± 1.94) (Figures 1b,c,g; Table 2).

**Figure 1:**
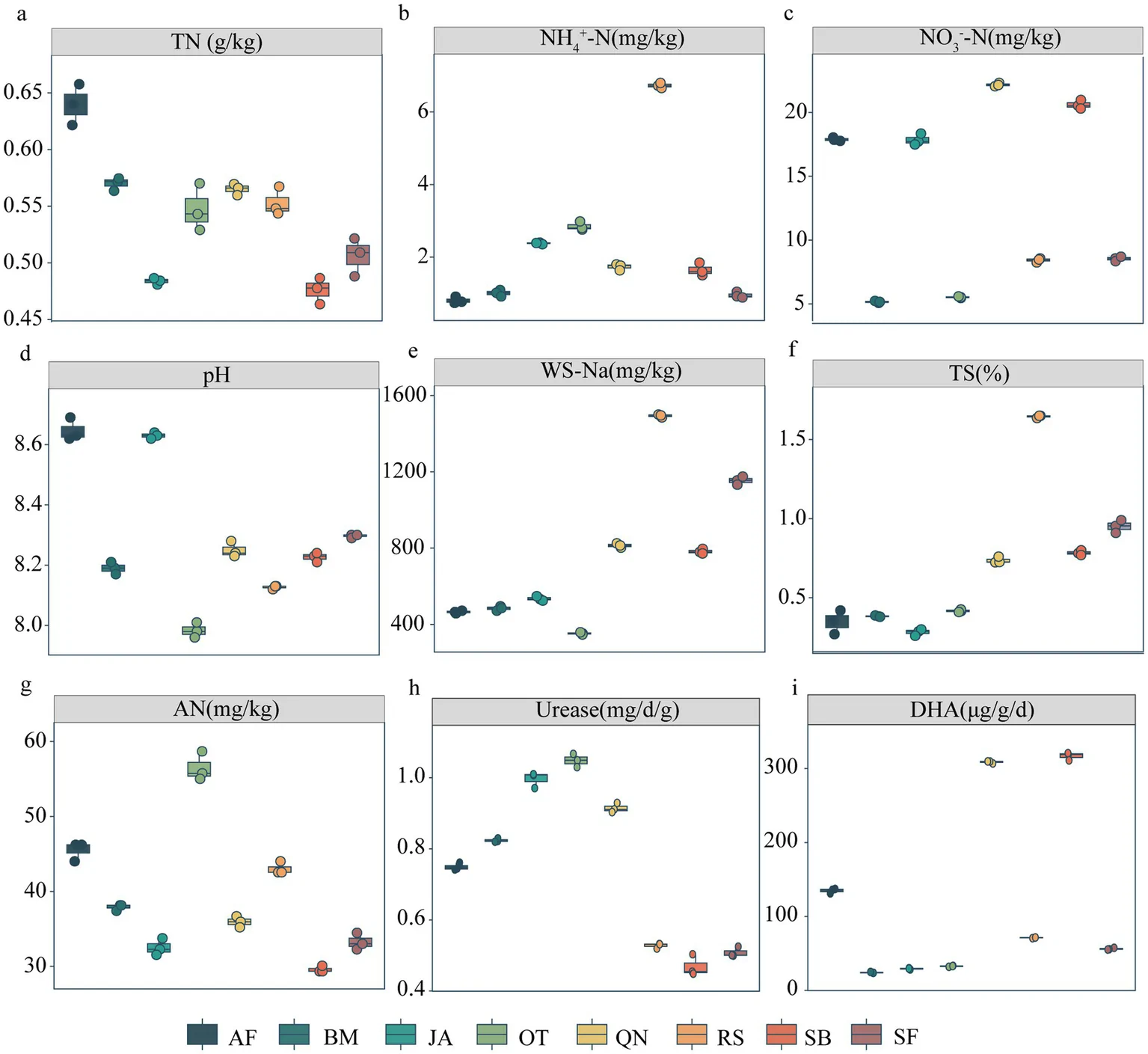
Rhizosphere soil properties and enzyme activities across eight crops. Panels show **(a)** total nitrogen (TN, g kg^−1^), **(b)** ammonium nitrogen (NH₄^+^–N, mg kg^−1^), **(c)** nitrate nitrogen (NO₃^−^–N, mg kg^−1^), **(d)** soil pH, **(e)** water-soluble sodium (WS-Na, mg kg^−1^), **(f)** total salt content (TS, %), **(g)** alkali-hydrolysable nitrogen (AN, mg kg^−1^), **(h)** urease activity (Urease, mg d^−1^ g^−1^), and **(i)** dehydrogenase activity (DHA, μg g^−1^ d^−1^). Boxplots show the median and interquartile range, and individual points represent the three biological replicates. Crop abbreviations: AF, alfalfa; BM, broomcorn millet; JA, Jerusalem artichoke; OT, oat; QN, quinoa; RS, rapeseed; SB, sugar beet; SF, sunflower.

**Table 2:** Physicochemical and enzymatic properties of rhizospheric soil across eight different crop species.

Group	TN (g kg⁻^1^)	OC (g kg⁻^1^)	TP (g kg⁻^1^)	TK (g kg⁻^1^)	NH₄⁺–N (mg kg⁻^1^)	NO₃⁻–N (mg kg⁻^1^)	AN (mg kg⁻^1^)	Urease (mg d⁻^1^ g⁻^1^)	DHA (μg g⁻^1^ d⁻^1^)	pH	WS-Na (mg kg⁻^1^)	TS (%)	MC (%)
AF	0.64 ± 0.02ᵃ	9.98 ± 0.24ᵃ	0.82 ± 0.02ᵃ	20.48 ± 0.18ᵇ^,^ᶜ	0.81 ± 0.09ᵉ	17.89 ± 0.14ᶜ	45.49 ± 1.27ᵇ	0.75 ± 0.01ᵉ	134.85 ± 3.22ᶜ	8.65 ± 0.04ᵃ	465.00 ± 7.00ᶠ	0.35 ± 0.08ᵉᶠ	7.23 ± 0.01ᶠ
BM	0.57 ± 0.01ᵇ	8.61 ± 0.25ᵇ	0.56 ± 0.01ᵉ	21.12 ± 0.60ᵃ^,^ᵇ	1.01 ± 0.09ᵉ	5.16 ± 0.08ᵉ	37.90 ± 0.43ᵈ	0.82 ± 0.01ᵈ	24.05 ± 0.76ᵍ	8.19 ± 0.02ᵈ	484.33 ± 11.59ᶠ	0.38 ± 0.01ᵈᵉ	9.38 ± 0.03ᶜ
JA	0.48 ± 0.01ᶜ^,^ᵈ	5.52 ± 0.11ᶠ	0.58 ± 0.01ᵉ	20.64 ± 0.17ᵃ^,^ᵇ^,^ᶜ	2.38 ± 0.02ᶜ	17.86 ± 0.44ᶜ	32.52 ± 1.12ᵉ	1.00 ± 0.02ᵇ	29.21 ± 0.87ᶠ	8.63 ± 0.01ᵃ	535.17 ± 11.73ᵉ	0.28 ± 0.02ᶠ	6.64 ± 0.14ᵍ
OT	0.55 ± 0.02ᵇ	7.88 ± 0.14ᶜ	0.76 ± 0.02ᶜ	20.50 ± 0.32ᵇ^,^ᶜ	2.85 ± 0.12ᵇ	5.53 ± 0.06ᵉ	56.49 ± 1.94ᵃ	1.05 ± 0.02ᵃ	32.64 ± 0.70ᶠ	7.98 ± 0.03ᶠ	352.83 ± 6.01ᵍ	0.42 ± 0.01ᵈ	6.56 ± 0.01ᵍ
QN	0.57 ± 0.01ᵇ	7.89 ± 0.26ᶜ	0.61 ± 0.01ᵈ	20.88 ± 0.12ᵃ^,^ᵇ^,^ᶜ	1.74 ± 0.09ᵈ	22.17 ± 0.13ᵃ	35.95 ± 0.74ᵈ	0.91 ± 0.02ᶜ	308.85 ± 1.57ᵇ	8.25 ± 0.03ᶜ	813.83 ± 11.25ᶜ	0.74 ± 0.02ᶜ	7.73 ± 0.03ᵉ
RS	0.55 ± 0.02ᵇ	7.74 ± 0.15ᶜ^,^ᵈ	0.80 ± 0.01ᵇ	21.26 ± 0.51ᵃ	6.73 ± 0.07ᵃ	8.43 ± 0.16ᵈ	43.04 ± 0.85ᶜ	0.53 ± 0.01ᶠ	71.17 ± 0.50ᵈ	8.13 ± 0.01ᵉ	1493.67 ± 7.77ᵃ	1.65 ± 0.01ᵃ	13.54 ± 0.04ᵃ
SB	0.48 ± 0.02ᵈ	6.97 ± 0.19ᵉ	0.53 ± 0.01ᶠ	20.23 ± 0.28ᶜ	1.65 ± 0.18ᵈ	20.61 ± 0.34ᵇ	29.59 ± 0.43ᶠ	0.47 ± 0.03ᵍ	317.10 ± 5.24ᵃ	8.23 ± 0.02ᶜ^,^ᵈ	782.67 ± 12.22ᵈ	0.78 ± 0.02ᶜ	7.99 ± 0.07ᵈ
SF	0.51 ± 0.02ᶜ	7.49 ± 0.12ᵈ	0.54 ± 0.01ᶠ	21.06 ± 0.40ᵃ^,^ᵇ	0.95 ± 0.09ᵉ	8.54 ± 0.18ᵈ	33.26 ± 1.12ᵉ	0.51 ± 0.01ᶠ	56.12 ± 1.23ᵉ	8.30 ± 0.01ᵇ	1154.17 ± 21.26ᵇ	0.95 ± 0.04ᵇ	12.52 ± 0.14ᵇ

Data are presented as means ± standard deviation (SD) of three biological replicates (*n* = 3). Different lowercase superscript letters within the same column indicate significant differences among crop species at *p* < 0.05 according to Tukey’s HSD test. Crop abbreviations: AF, alfalfa (*Medicago sativa* L.); BM, broomcorn millet (*Panicum miliaceum* L.); JA, Jerusalem artichoke (*Helianthus tuberosus* L.); OT, oat (*Avena sativa* L.); QN, quinoa (*Chenopodium quinoa* Willd.); RS, rapeseed (*Brassica napus* L.); SB, sugar beet (*Beta vulgaris* L.); SF, sunflower (*Helianthus annuus* L.). Parameter abbreviations: TN, total nitrogen; OC, organic carbon; TP, total phosphorus; TK, total potassium; NH₄⁺–N, ammonium nitrogen; NO₃⁻–N, nitrate nitrogen; AN, alkali-hydrolysable nitrogen; Urease, soil urease activity; DHA, dehydrogenase activity; WS-Na, water-soluble sodium; TS, total salt content; MC, soil moisture content.

Salinity- and moisture-related traits showed similarly distinct crop-associated patterns. Rapeseed had the highest water-soluble Na^+^ (WS-Na), total soluble salts (TS) and moisture content (MC), reaching 1493.67 ± 7.77, 1.65 ± 0.01 and 13.54 ± 0.04, respectively, while sunflower showed the second-highest values for these traits (Figures 1e,f; Supplementary Figure S1d; Table 2). Oat had the lowest WS-Na concentration (352.83 ± 6.01), and Jerusalem artichoke had the lowest TS content (0.28 ± 0.02). Soil pH remained alkaline across all crop rhizospheres, ranging from 7.98 ± 0.03 in oat to 8.65 ± 0.04 in alfalfa (Figure 1d). Enzyme activities also differed among crops: urease activity was highest in oat (1.05 ± 0.02), whereas dehydrogenase activity was highest in sugar beet (317.10 ± 5.24) (Figures 1h,i; Table 2).

Overall, crop identity was associated with coordinated differences in rhizosphere nutrient status, N forms, salinity–moisture conditions and enzyme activities.

### Plant nutrient accumulation and Na^+^–K^+^ allocation differed among crops

Aboveground nutrient and ion traits differed markedly among crops (Supplementary Figures S2a–d). Quinoa had the highest aboveground TN and TK, while oat showed the highest TP. Aboveground Na^+^ and Na^+^/K^+^ ratios also varied substantially (Figure 2a): sugar beet and oat showed relatively high values, whereas quinoa maintained a low Na^+^/K^+^ ratio primarily because of its high K^+^ concentration.

**Figure 2:**
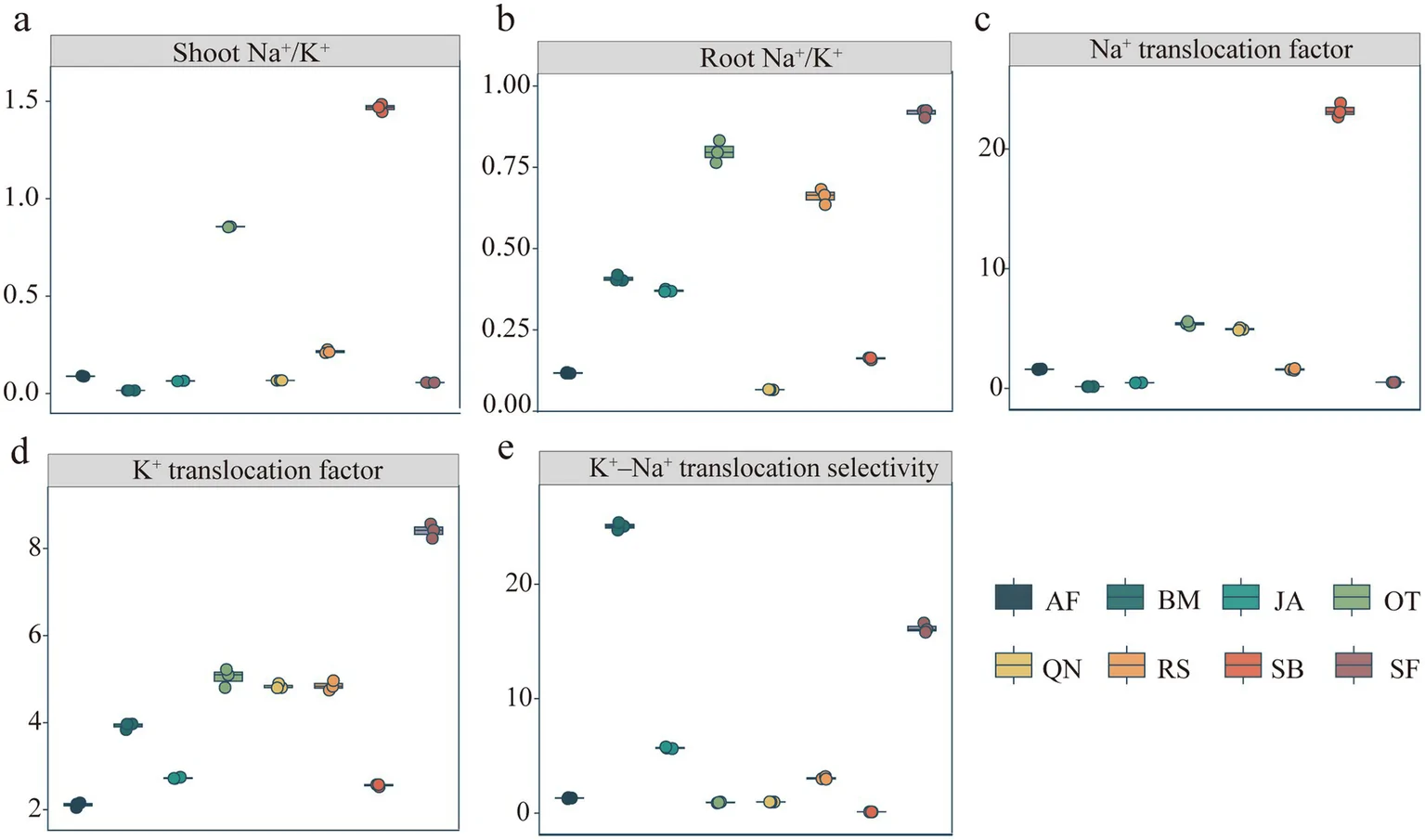
Plant Na^+^–K^+^ balance and ion translocation across eight crops. Panels show **(a)** shoot Na^+^/K^+^ ratio, **(b)** root Na^+^/K^+^ ratio, **(c)** Na^+^ translocation factor, calculated as the ratio of shoot Na^+^ concentration to root Na^+^ concentration, **(d)** K^+^ translocation factor, calculated as the ratio of shoot K^+^ concentration to root K^+^ concentration, and **(e)** K^+^–Na^+^ translocation selectivity, calculated as the shoot K^+^/Na^+^ ratio divided by the root K^+^/Na^+^ ratio. Boxplots show the median and interquartile range, and individual points represent three biological replicates.

Root traits showed distinct crop-specific profiles (Supplementary Figures S2e–h; Figure 2b). Quinoa had the highest root TK and the lowest root Na^+^/K^+^ ratio, whereas sunflower showed the highest root Na^+^ concentration and one of the highest root Na^+^/K^+^ ratios. These differences indicate marked variation in root Na^+^–K^+^ composition among crops.

Na^+^ and K^+^ translocation indices further differentiated ion allocation among crops (Figures 2c–e). Sugar beet had the highest Na^+^ translocation factor, whereas broomcorn millet, Jerusalem artichoke and sunflower showed relatively low Na^+^ translocation. Sunflower combined high root Na^+^ with low Na^+^ translocation and high K^+^ translocation, indicating a predominantly root-associated Na^+^ distribution together with greater K^+^ allocation to aboveground tissues. Overall, quinoa was characterized by high K^+^ concentrations and low Na^+^/K^+^ ratios, sunflower by root-associated Na^+^ distribution and strong K^+^ translocation, and sugar beet by high aboveground Na^+^ and Na^+^ translocation, highlighting distinct crop-specific Na^+^–K^+^ allocation characteristics.

### Crop identity strongly structured bacterial and fungal diversity

The Shannon-Wiener rarefaction curves of both bacterial and fungal communities gradually approached a plateau, indicating that the sequencing depth was sufficient to capture the major microbial diversity in the rhizosphere samples (Supplementary Figure S3).

Crop identity significantly affected bacterial and fungal alpha diversity, with all six indices remaining significant after FDR correction (Supplementary Figure S4; Table 3). Dunn’s *post hoc* tests with Benjamini–Hochberg adjustment showed that Jerusalem artichoke had significantly higher bacterial Chao1 richness and Shannon diversity than rapeseed, and bacterial phylogenetic diversity was higher in both Jerusalem artichoke and sunflower than in rapeseed. For fungi, Shannon diversity was significantly higher in broomcorn millet and sunflower than in sugar beet. Although overall crop effects were also significant for fungal Chao1 richness and Faith’s PD, no individual pairwise comparison remained significant after multiple-comparison correction.

**Table 3:** Kruskal–Wallis and Dunn’s *post hoc* comparisons of bacterial and fungal alpha diversity among the eight crops.

Part A					
Community	Metric	Kruskal–Wallis *H*	df	Raw *P*	BH-adjusted *P*
Bacteria (16S)	Chao1	17.787	7	0.013	0.019
Bacteria (16S)	Shannon	19.217	7	0.008	0.019
Bacteria (16S)	Faith’s PD	19.200	7	0.008	0.019
Fungi (ITS)	Chao1	17.027	7	0.017	0.021
Fungi (ITS)	Shannon	18.613	7	0.009	0.019
Fungi (ITS)	Faith’s PD	16.253	7	0.023	0.023

Part B						
Community	Metric	Group 1	Group 2	Z	Raw *p*	BH-adjusted *p*
Bacteria (16S)	Chao1	JA	RS	−3.2332	0.0012	0.0343
Bacteria (16S)	Shannon	JA	RS	−3.4656	0.0005	0.0148
Bacteria (16S)	Faith’s PD	JA	RS	−3.2332	0.0012	0.0171
Bacteria (16S)	Faith’s PD	RS	SF	3.2332	0.0012	0.0171
Fungi (ITS)	Shannon	BM	SB	−3.3486	0.0008	0.0227
Fungi (ITS)	Shannon	SB	SF	3.0022	0.0027	0.0375

Part A presents the global Kruskal–Wallis test results, and Part B presents significant Dunn pairwise comparisons. *p* values from the six global alpha-diversity tests were adjusted using the Benjamini–Hochberg false discovery rate (FDR) procedure. For indices with a significant overall crop effect, Dunn’s *post hoc* tests were conducted, with Benjamini–Hochberg adjustment applied to pairwise comparisons within each index. Only pairwise comparisons with adjusted *p* < 0.05 are shown. H, Kruskal–Wallis test statistic; Z, Dunn test statistic; FDR, false discovery rate.

Beta diversity analysis further confirmed the strong effect of crop identity on microbial community structure (Supplementary Figures S5a,b). PERMANOVA based on Bray–Curtis distances showed that crop identity explained 78.1% of bacterial community variation (*R^2^* = 0.781, *p* = 0.001) and 83.5% of fungal community variation (*R^2^* = 0.835, *p* = 0.001). PCoA ordinations showed clear clustering of both bacterial and fungal communities according to crop species. The higher *R^2^* value for fungi suggests that fungal community composition was slightly more strongly structured by crop identity than bacterial community composition. UpSet analysis showed that bacterial communities had a larger shared core, whereas fungal communities showed greater crop specificity (Supplementary Figures S5c,d). For bacteria, 1,188 OTUs were shared across all crop rhizospheres, and Jerusalem artichoke had the largest number of unique bacterial OTUs, whereas rapeseed had the fewest unique bacterial OTUs. For fungi, only 87 OTUs were shared across all crop rhizospheres, and sunflower had the highest number of unique fungal OTUs, whereas rapeseed had the fewest.

Taken together, crop identity strongly structured rhizosphere microbial diversity and community composition. Bacterial communities contained a larger shared OTU core, while fungal communities had fewer shared OTUs and greater crop-specific differentiation. These results indicate that bacterial and fungal communities responded differently to crop identity under saline-alkaline conditions.

### Crop-specific microbial taxa and environment-responsive OTUs linked community composition to rhizosphere conditions

At the phylum level, bacterial communities were dominated mainly by Actinobacteriota and Proteobacteria, followed by Chloroflexi, Bacteroidota, Gemmatimonadota, Acidobacteriota, Myxococcota, Planctomycetota, Verrucomicrobiota and Patescibacteria (Figure 3a). Although these dominant phyla occurred in all crop rhizospheres, their relative abundances varied among crops. Fungal communities were dominated by Ascomycota across all crop rhizospheres, with Basidiomycota, Mortierellomycota, Chytridiomycota and other fungal phyla contributing smaller but variable proportions (Figure 3b).

**Figure 3:**
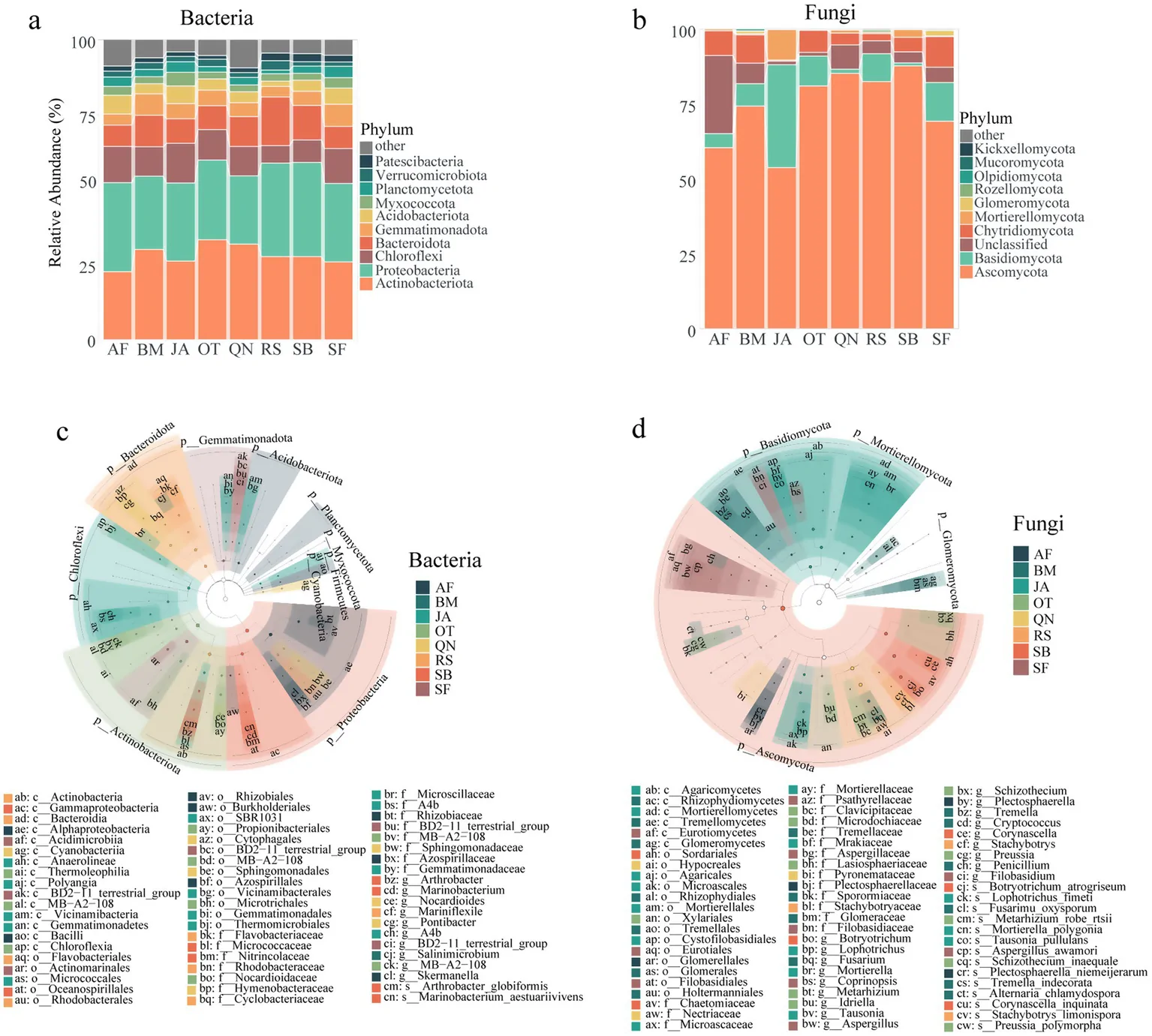
Crop-associated bacterial and fungal taxonomic composition and differential lineages. Stacked bar plots show the relative abundances of dominant bacterial **(a)** and fungal **(b)** phyla across the eight crop rhizospheres. Taxa with relatively low abundance are grouped as “other.” Circular linear discriminant analysis effect size (LEfSe) cladograms show bacterial **(c)** and fungal **(d)** taxonomic lineages that differed among crops. Concentric taxonomic levels extend from higher to lower ranks, and colored sectors and nodes indicate lineages enriched in the corresponding crop rhizosphere. Differential taxa were identified using a linear discriminant analysis (LDA) score > 3.5 and *p* < 0.05.

At finer taxonomic levels, bacterial communities were mainly composed of Alphaproteobacteria, Actinobacteria, Acidimicrobiia, Gammaproteobacteria, Bacteroidia and Thermoleophilia at the class level (Supplementary Figure S6). Dominant bacterial orders included Rhizobiales, Actinomarinales, Micrococcales, Cytophagales, Microtrichales and Flavobacteriales. Fungal communities were dominated by Sordariomycetes and Dothideomycetes at the class level, while Hypocreales, Pleosporales, Sordariales, Agaricales, Microascales and Mortierellales were the major fungal orders. Compared with bacteria, fungal taxa showed clearer crop-specific differentiation at several taxonomic ranks.

LEfSe analysis identified distinct crop-specific bacterial and fungal biomarkers (Figures 3c,d). Differential bacterial taxa were mainly affiliated with Actinobacteriota, Proteobacteria, Chloroflexi, Bacteroidota, Acidobacteriota and Gemmatimonadota, while differential fungal taxa were mainly affiliated with Ascomycota, Basidiomycota, Mortierellomycota and Glomeromycota. These biomarkers occurred across multiple taxonomic levels, indicating that crop identity selected distinct microbial lineages rather than only shifting broad phylum-level composition.

RDA revealed distinct environmental associations underlying bacterial and fungal community differentiation (Figures 4a,b). The first two axes accounted for 31.43% and 31.04% of the bacterial and fungal ordination variation, respectively. Bacterial community structure was significantly associated with a broad set of rhizosphere variables, including TN, OC, TP, AN, pH, urease activity, total salts and soil moisture, indicating that bacterial differentiation was associated with combined gradients in nutrient status, salinity and alkalinity. In contrast, fungal community variation was more strongly associated with soil moisture and nutrient-related variables, particularly TN, OC and TK. These contrasting associations suggest that bacterial and fungal communities responded to different components of the crop-modified rhizosphere environment.

**Figure 4:**
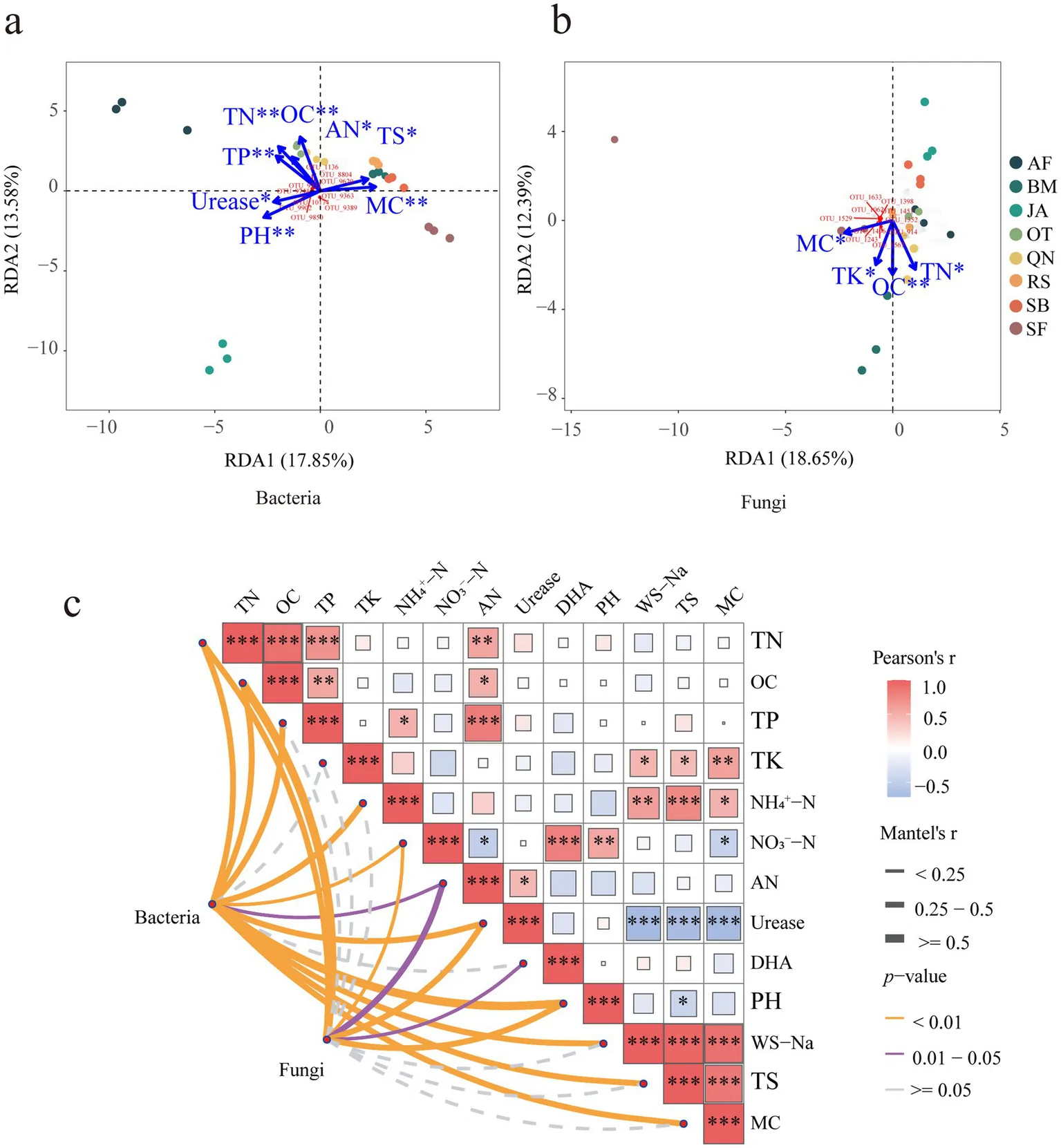
Associations between rhizosphere microbial communities and environmental variables. Redundancy analysis (RDA) ordinations show associations of rhizosphere environmental variables with bacterial **(a)** and fungal **(b)** community composition. RDA1 and RDA2 explained 17.85 and 13.58% of the bacterial ordination variation and 18.65 and 12.39% of the fungal ordination variation, respectively. Colored points represent biological replicates of the eight crops. Environmental vectors are shown as arrows, with significant fitted variables highlighted in blue; red labels indicate the top 10 environment-responsive operational taxonomic units (OTUs) identified by permutation-based vector fitting. Asterisks associated with environmental vectors indicate significance (*p* < 0.05; *p* < 0.01). **(c)** Integrated Pearson correlation and Mantel test analysis. Cell color represents the direction and magnitude of Pearson’s r among environmental variables, whereas line thickness represents the magnitude of Mantel’s r between microbial community dissimilarity and individual environmental variables (< 0.25, 0.25–0.5, or ≥ 0.5). Line color indicates Mantel test significance (*p* < 0.01, 0.01–0.05, or ≥ 0.05). Asterisks in the correlation matrix denote Pearson correlation significance (*p* < 0.05, *p* < 0.01, and *p* < 0.001). OC, organic carbon; TP, total phosphorus; TK, total potassium; MC, soil moisture content.

Environment-responsive OTUs provided taxon-level evidence for these environmental associations (Figures 4a,b; Table 4). The top 10 bacterial OTUs most responsive to environmental variables had *r* values ranging from 0.806 to 0.879, with *p* = 0.001 and FDR = 0.014. These OTUs were affiliated with Proteobacteria, Actinobacteriota, Gemmatimonadota, Chloroflexi and Bacteroidota, including taxa such as *Skermanella*, *TX1A-55*, *Erythrobacter* and *Echinicola*. The top 10 fungal OTUs showed even stronger correlations, with *r* values ranging from 0.933 to 0.967, *p* = 0.001 and FDR = 0.021. These fungal OTUs were affiliated with Ascomycota, Basidiomycota and Mortierellomycota, including *Botryotrichum, Mrakia, Psathyrella, Mortierella* and *Lophotrichus*.

**Table 4:** Taxonomic classification and RDA scores of the top 10 bacterial and top 10 fungal OTUs most responsive to environmental variables.

OTU	RDA1	RDA2	*r*	*P*	FDR	Phylum	Class	Order	Family	Genus	Species
OTU_9332	−0.057	0.031	0.879	0.001	0.014	Proteobacteria	Alphaproteobacteria	Azospirillales	Azospirillaceae	*Skermanella*	uncultured_bacterium
OTU_1606	0.037	−0.035	0.874	0.001	0.014	Proteobacteria	Gammaproteobacteria	Acidithiobacillales	Acidithiobacillaceae	*TX1A-55*	uncultured_gamma
OTU_8164	0.109	−0.058	0.860	0.001	0.014	Actinobacteriota	Acidimicrobiia	Actinomarinales	uncultured	uncultured	bacterium_YC-LK-LKJ36
OTU_318	0.026	−0.020	0.838	0.001	0.014	Gemmatimonadota	BD2-11_terrestrial_group	BD2-11_terrestrial_group	BD2-11_terrestrial_group	*BD2-11_terrestrial_group*	uncultured_bacterium
OTU_1620	0.046	−0.042	0.836	0.001	0.014	Proteobacteria	Gammaproteobacteria	Gammaproteobacteria_Incertae_Sedis	Unknown_Family	uncultured	uncultured_bacterium
OTU_8044	0.014	−0.029	0.827	0.001	0.014	Chloroflexi	OLB14	OLB14	OLB14	*OLB14*	uncultured_bacterium
OTU_1711	0.033	−0.034	0.819	0.001	0.014	Proteobacteria	Gammaproteobacteria	EPR3968-O8a-Bc78	EPR3968-O8a-Bc78	*EPR3968-O8a-Bc78*	uncultured_gamma
OTU_6875	0.039	0.015	0.813	0.001	0.014	Actinobacteriota	Actinobacteria	Nitriliruptorales	Nitriliruptoraceae	*Nitriliruptoraceae*	uncultured_bacterium
OTU_9450	0.046	0.037	0.807	0.001	0.014	Proteobacteria	Alphaproteobacteria	Sphingomonadales	Sphingomonadaceae	*Erythrobacter*	Erythrobacter_sp.
OTU_5208	0.072	−0.020	0.806	0.001	0.014	Bacteroidota	Bacteroidia	Cytophagales	Cyclobacteriaceae	*Echinicola*	Echinicola_sediminis
OTU_751	0.127	0.628	0.967	0.001	0.021	Ascomycota	Sordariomycetes	Sordariales	Chaetomiaceae	*Botryotrichum*	Botryotrichum_atrogriseum
OTU_1059	0.008	−0.003	0.960	0.001	0.021	Basidiomycota	Tremellomycetes	Cystofilobasidiales	Mrakiaceae	*Mrakia*	Mrakia_blollopis
OTU_196	0.007	−0.002	0.953	0.001	0.021	Basidiomycota	Agaricomycetes	Agaricales	Psathyrellaceae	*Psathyrella*	Psathyrella_canoceps
OTU_954	0.162	−0.063	0.944	0.001	0.021	Mortierellomycota	Mortierellomycetes	Mortierellales	Mortierellaceae	*Mortierella*	Mortierella_polygonia
OTU_808	0.122	−0.047	0.942	0.001	0.021	Mortierellomycota	Mortierellomycetes	Mortierellales	Mortierellaceae	*Mortierella*	Mortierella_polygonia
OTU_218	0.428	−0.159	0.940	0.001	0.021	Basidiomycota	Agaricomycetes	Agaricales	unidentified	unidentified	unidentified
OTU_965	0.101	−0.033	0.938	0.001	0.021	Mortierellomycota	Mortierellomycetes	Mortierellales	Mortierellaceae	*Mortierella*	Mortierella_indohii
OTU_579	0.018	−0.007	0.937	0.001	0.021	Ascomycota	unidentified	unidentified	unidentified	unidentified	unidentified
OTU_1208	0.007	−0.003	0.934	0.001	0.021	Ascomycota	Sordariomycetes	Microascales	Microascaceae	*Lophotrichus*	Lophotrichus_fimeti
OTU_898	0.098	−0.036	0.933	0.001	0.021	Mortierellomycota	Mortierellomycetes	Mortierellales	Mortierellaceae	*Mortierella*	Mortierella_indohii

RDA1 and RDA2 are species coordinates extracted from the fitted RDA model based on the Hellinger-transformed community matrix and constrained by the measured environmental variables. OTUs were ranked according to their goodness of fit to the RDA ordination using permutation-based vector fitting (envfit; 999 permutations), and the top 10 bacterial and top 10 fungal OTUs were selected accordingly. Taxonomic information (phylum to species) was assigned following the annotation pipeline described in the Methods; entries reported as “uncultured” or “unidentified” indicate that confident classification at the corresponding rank was not available.

Pearson correlation analysis revealed significant covariation among several rhizosphere properties (Figure 4c). TN was positively correlated with OC, TP and AN, whereas WS-Na, total salts and soil moisture were strongly positively correlated with each other. Urease activity was negatively correlated with these salinity-related variables. Mantel tests further showed that bacterial and fungal community dissimilarities were significantly associated with multiple rhizosphere variables, indicating that microbial community differentiation was related to coordinated changes in soil nutrient status, salinity, moisture and biochemical activity.

Together, these results indicate that crop-specific microbial communities were shaped by both crop identity and rhizosphere environmental gradients. Bacterial communities were dominated by Actinobacteriota and Proteobacteria, while fungal communities were dominated by Ascomycota, but LEfSe analysis revealed crop-specific biomarkers within these broad dominant lineages. RDA, environment-responsive OTUs and correlation analyses further showed that soil nutrient status, salt-water conditions and enzyme activity were closely associated with microbial community differentiation.

### Rhizosphere microbial network organization differed among crops

Co-occurrence network analysis showed that bacterial nodes dominated the microbial networks across all crop rhizospheres (Supplementary Figure S7; Table 5). Bacterial nodes accounted for 79.39%–85.14% of total nodes, whereas fungal nodes accounted for 14.70%–20.60%. Sugar beet had the highest bacterial node proportion, while broomcorn millet and quinoa had the highest fungal node proportions. This indicates that bacterial taxa formed the main structural component of the rhizosphere microbial networks, although the fungal contribution varied among crops.

**Table 5:** Taxonomic composition and the relative proportions of the top five modules in the co-occurrence networks across the rhizosphere of eight crops.

Group	Bacteria	Fungi	Module 1	Module 2	Module 3	Module 4	Module 5
AF	84.89%	14.96%	26.72%	12.06%	11.45%	8.24%	7.33%
BM	79.40%	20.60%	16.94%	14.45%	13.12%	9.47%	8.97%
JA	84.53%	15.31%	20.31%	18.28%	16.25%	11.56%	10.63%
OT	83.71%	16.14%	17.19%	14.18%	13.27%	13.12%	7.24%
QN	79.39%	20.60%	18.75%	14.86%	11.66%	9.63%	9.12%
RS	83.13%	16.87%	20.06%	19.60%	15.50%	13.07%	9.73%
SB	85.14%	14.70%	19.49%	18.37%	16.77%	13.74%	10.38%
SF	82.22%	17.78%	20.82%	16.87%	15.35%	15.20%	9.73%

The proportions of bacteria and fungi represent the percentage of total nodes belonging to each kingdom within the respective co-occurrence networks. The top five modules refer to the five largest clusters of highly interconnected nodes, ranked by their relative size (number of nodes).

Network topology revealed that connectedness and complexity represented different aspects of microbial organization (Table 6). Oat had the largest number of nodes (663), followed by rapeseed and sunflower, each with 658 nodes. Sunflower had the highest number of edges (4247), average degree (12.909) and network density (0.020), followed closely by rapeseed, which had 4,202 edges, an average degree of 12.772 and a network density of 0.019. In contrast, broomcorn millet and quinoa had the fewest edges and lowest average degree values. However, network complexity was highest in Jerusalem artichoke (0.568), followed by oat (0.563), sugar beet (0.556) and alfalfa (0.550), whereas broomcorn millet and quinoa had the lowest complexity values. Therefore, sunflower and rapeseed had the most densely connected networks, while Jerusalem artichoke and oat showed higher overall network complexity (Supplementary Figure S7).

**Table 6:** Topological properties of microbial co-occurrence networks in the rhizosphere of eight crop species.

Group	AF	BM	JA	OT	QN	RS	SB	SF
Number of nodes	655	602	640	663	592	658	626	658
Number of edges	2,907	2071	3,687	2,953	2061	4,202	3,604	4,247
Positive edges	1,614	1,099	2050	1,650	1,091	2,327	1967	2,376
Negative edges	1,293	972	1,637	1,303	970	1875	1,637	1871
Average degree	8.876	6.880	11.522	8.908	6.963	12.772	11.514	12.909
Average path length	6.748	7.185	6.652	6.742	7.186	6.308	6.659	6.304
Network diameter	18.722	18.724	19.708	18.722	18.724	17.735	19.708	17.735
Network density	0.014	0.011	0.018	0.013	0.012	0.019	0.018	0.020
Average clustering coefficient	0.628	0.635	0.614	0.628	0.635	0.608	0.614	0.608
Degree centralization	0.026	0.020	0.024	0.026	0.02	0.026	0.025	0.026
Betweenness centralization	0.025	0.039	0.027	0.024	0.04	0.027	0.028	0.027
Modularity	0.748	0.804	0.750	0.758	0.804	0.715	0.743	0.721
Number of keystone nodes	13	3	5	13	5	2	6	3
Maximum information centrality	0.296	0.307	0.165	0.296	0.307	0.093	0.166	0.094
Network complexity	0.550	0.404	0.568	0.563	0.405	0.500	0.556	0.510

Module composition showed that Proteobacteria and Actinobacteriota were central to network organization (Supplementary Figure S8; Table 7). Across major modules, dominant phyla were mainly Proteobacteria, Actinobacteriota, Ascomycota, Chloroflexi and Bacteroidota. Proteobacteria and Actinobacteriota were the dominant bacterial phyla across most modules. For example, all five major modules in Jerusalem artichoke were dominated by Proteobacteria, and Proteobacteria also ranked first in all five major modules in sugar beet. Keystone analysis showed that connector nodes were detected in all crop networks, whereas module hubs were detected only in Jerusalem artichoke, oat and sunflower (Table 8). The identified module hubs were OTU_377 in Jerusalem artichoke, OTU_546 in oat and OTU_1024 in sunflower.

**Table 7:** Distribution and proportions of the top three dominant bacterial and fungal phyla within the five largest modules of the co-occurrence networks.

Group	Rank	Module 1	Count	Proportion (%)	Module 2	Count	Proportion (%)	Module 3	Count	Proportion (%)	Module 4	Count	Proportion (%)	Module 5	Count	Proportion (%)
AF	1	Actinobacteriota	41	23.4%	Proteobacteria	16	20.3%	Proteobacteria	20	26.7%	Proteobacteria	19	35.2%	Actinobacteriota	13	27.1%
AF	2	Proteobacteria	41	23.4%	Actinobacteriota	12	15.2%	Actinobacteriota	16	21.3%	Actinobacteriota	10	18.5%	Proteobacteria	9	18.8%
AF	3	Ascomycota	22	12.6%	Ascomycota	9	11.4%	Chloroflexi	11	14.7%	Ascomycota	4	7.4%	Acidobacteriota	6	12.5%
BM	1	Actinobacteriota	23	22.5%	Proteobacteria	22	25.3%	Proteobacteria	19	24.1%	Actinobacteriota	13	22.8%	Proteobacteria	13	24.1%
BM	2	Proteobacteria	21	20.6%	Actinobacteriota	16	18.4%	Actinobacteriota	14	17.7%	Ascomycota	10	17.5%	Actinobacteriota	11	20.4%
BM	3	Bacteroidota	16	15.7%	Chloroflexi	9	10.3%	Bacteroidota	11	13.9%	Proteobacteria	10	17.5%	Chloroflexi	5	9.3%
JA	1	Proteobacteria	42	32.3%	Proteobacteria	30	25.6%	Proteobacteria	31	29.8%	Proteobacteria	22	29.7%	Proteobacteria	16	23.5%
JA	2	Actinobacteriota	26	20.0%	Actinobacteriota	19	16.2%	Actinobacteriota	20	19.2%	Actinobacteriota	15	20.3%	Actinobacteriota	10	14.7%
JA	3	Chloroflexi	18	13.8%	Ascomycota	17	14.5%	Chloroflexi	11	10.6%	Ascomycota	8	10.8%	Ascomycota	10	14.7%
OT	1	Actinobacteriota	32	28.1%	Proteobacteria	25	26.6%	Proteobacteria	27	30.7%	Proteobacteria	20	23.0%	Actinobacteriota	13	27.1%
OT	2	Proteobacteria	24	21.1%	Actinobacteriota	21	22.3%	Actinobacteriota	13	14.8%	Actinobacteriota	13	14.9%	Proteobacteria	9	18.8%
OT	3	Chloroflexi	16	14.0%	Ascomycota	11	11.7%	Ascomycota	9	10.2%	Ascomycota	11	12.6%	Acidobacteriota	6	12.5%
QN	1	Proteobacteria	27	24.3%	Proteobacteria	20	22.7%	Bacteroidota	14	20.3%	Actinobacteriota	13	22.8%	Proteobacteria	13	24.1%
QN	2	Actinobacteriota	22	19.8%	Actinobacteriota	18	20.5%	Proteobacteria	14	20.3%	Proteobacteria	11	19.3%	Actinobacteriota	11	20.4%
QN	3	Bacteroidota	16	14.4%	Ascomycota	11	12.5%	Actinobacteriota	13	18.8%	Ascomycota	10	17.5%	Chloroflexi	5	9.3%
RS	1	Proteobacteria	38	28.8%	Proteobacteria	35	27.1%	Actinobacteriota	28	27.5%	Proteobacteria	25	29.1%	Actinobacteriota	19	29.7%
RS	2	Actinobacteriota	35	26.5%	Chloroflexi	19	14.7%	Proteobacteria	25	24.5%	Actinobacteriota	12	14.0%	Proteobacteria	10	15.6%
RS	3	Ascomycota	14	10.6%	Actinobacteriota	18	14.0%	Ascomycota	8	7.8%	Chloroflexi	11	12.8%	Bacteroidota	9	14.1%
SB	1	Proteobacteria	33	27.0%	Proteobacteria	33	28.7%	Proteobacteria	34	32.4%	Proteobacteria	27	31.4%	Proteobacteria	14	21.5%
SB	2	Actinobacteriota	20	16.4%	Actinobacteriota	21	18.3%	Actinobacteriota	23	21.9%	Actinobacteriota	16	18.6%	Actinobacteriota	10	15.4%
SB	3	Ascomycota	17	13.9%	Chloroflexi	12	10.4%	Ascomycota	12	11.4%	Chloroflexi	15	17.4%	Ascomycota	10	15.4%
SF	1	Proteobacteria	40	29.2%	Proteobacteria	31	27.9%	Actinobacteriota	29	28.7%	Proteobacteria	30	30.0%	Actinobacteriota	18	28.1%
SF	2	Actinobacteriota	34	24.8%	Actinobacteriota	16	14.4%	Proteobacteria	18	17.8%	Actinobacteriota	15	15.0%	Proteobacteria	14	21.9%
SF	3	Bacteroidota	12	8.8%	Chloroflexi	15	13.5%	Ascomycota	12	11.9%	Chloroflexi	14	14.0%	Ascomycota	5	7.8%

Network modules were identified using the fast-greedy modularity optimization algorithm implemented in the igraph package. The top five modules (M1–M5) were ranked according to the number of nodes (OTUs) they contained. The top three phyla represent the three most abundant taxonomic groups at the phylum level within each module. Count refers to the number of OTUs belonging to a specific phylum within the module, and proportion (%) indicates the percentage of those OTUs relative to the total number of nodes in that module.

**Table 8:** The keystone taxa identified from the microbial co-occurrence networks across the eight groups.

Group	OTU	*Zi*	*Pi*	Topological role
AF	OTU_10253	0.000	1.000	Connector
AF	OTU_2268	2.134	0.623	Connector
AF	OTU_3470	2.134	0.623	Connector
AF	OTU_4835	−0.566	0.716	Connector
AF	OTU_4976	0.303	0.636	Connector
AF	OTU_60	0.126	0.660	Connector
AF	OTU_6875	0.000	1.000	Connector
AF	OTU_7141	1.284	0.643	Connector
AF	OTU_7584	0.000	1.000	Connector
AF	OTU_8684	0.303	0.636	Connector
AF	OTU_8743	0.303	0.636	Connector
AF	OTU_540	0.000	1.000	Connector
AF	OTU_812	−1.304	0.667	Connector
BM	OTU_1260	0.428	0.708	Connector
BM	OTU_1596	0.760	0.639	Connector
BM	OTU_9734	0.000	1.000	Connector
JA	OTU_1136	−0.026	0.625	Connector
JA	OTU_1728	0.045	0.722	Connector
JA	OTU_1739	0.045	0.722	Connector
JA	OTU_377	3.328	0.000	Module hub
JA	OTU_5679	0.000	1.000	Connector
OT	OTU_10371	0.308	0.667	Connector
OT	OTU_10848	0.308	0.667	Connector
OT	OTU_1758	0.000	1.000	Connector
OT	OTU_2268	2.134	0.623	Connector
OT	OTU_3470	2.134	0.623	Connector
OT	OTU_4835	−0.483	0.716	Connector
OT	OTU_60	0.126	0.660	Connector
OT	OTU_649	0.308	0.667	Connector
OT	OTU_7141	1.284	0.643	Connector
OT	OTU_8556	0.000	1.000	Connector
OT	OTU_8707	0.000	1.000	Connector
OT	OTU_9542	0.308	0.667	Connector
OT	OTU_546	2.713	0.000	Module hub
QN	OTU_10191	0.000	1.000	Connector
QN	OTU_1260	0.428	0.708	Connector
QN	OTU_1596	0.760	0.639	Connector
QN	OTU_6367	0.000	1.000	Connector
QN	OTU_603	0.000	1.000	Connector
RS	OTU_6040	0.000	1.000	Connector
RS	OTU_8390	0.000	1.000	Connector
SB	OTU_1136	−0.026	0.625	Connector
SB	OTU_1728	0.095	0.648	Connector
SB	OTU_1739	0.095	0.648	Connector
SB	OTU_6039	0.000	1.000	Connector
SB	OTU_6229	0.000	1.000	Connector
SB	OTU_8094	0.000	1.000	Connector
SF	OTU_5487	0.000	1.000	Connector
SF	OTU_6992	0.000	1.000	Connector
SF	OTU_1024	3.088	0.000	Module hub

Topological roles of nodes were categorized based on their Zi (within-module connectivity) and Pi (participation coefficient) values. Nodes with Zi > 2.5 and Pi < 0.62 were classified as module hubs, whereas nodes with Zi < 2.5 and Pi > 0.62 were classified as connectors.

As an exploratory analysis, PICRUSt2-based inference suggested variation in potential bacterial functional profiles among the crop-associated communities (Supplementary Figure S9). PCA of the inferred KEGG pathway profiles showed differences in their predicted functional composition, with PC1, PC2 and PC3 accounting for 38.8%, 20.0%, and 16.0% of the variation, respectively. The first three axes together explained 74.8% of the variation in the inferred profiles. Several predicted pathways associated with central carbon metabolism, amino acid metabolism, vitamin metabolism, antioxidant defence, DNA repair and bacterial chemotaxis contributed to these patterns. These results represent taxonomically inferred functional potential rather than experimentally measured pathway activity and were therefore used only to identify candidate processes for future validation.

### Integrated MFA classified the eight crops into stable rhizosphere ecological types

Integrated MFA separated the eight crops into two stable groups (Figure 5). Group I comprised Jerusalem artichoke, oat, alfalfa, broomcorn millet and quinoa, whereas Group II comprised sunflower, rapeseed and sugar beet. Representative-trait analysis showed that Group I was generally associated with lower rhizosphere salinity and relatively stronger nutrient- and enzyme-related characteristics, whereas Group II was characterized by higher rhizosphere salinity and greater contributions from Na^+^ allocation traits (Supplementary Figure S10).

**Figure 5:**
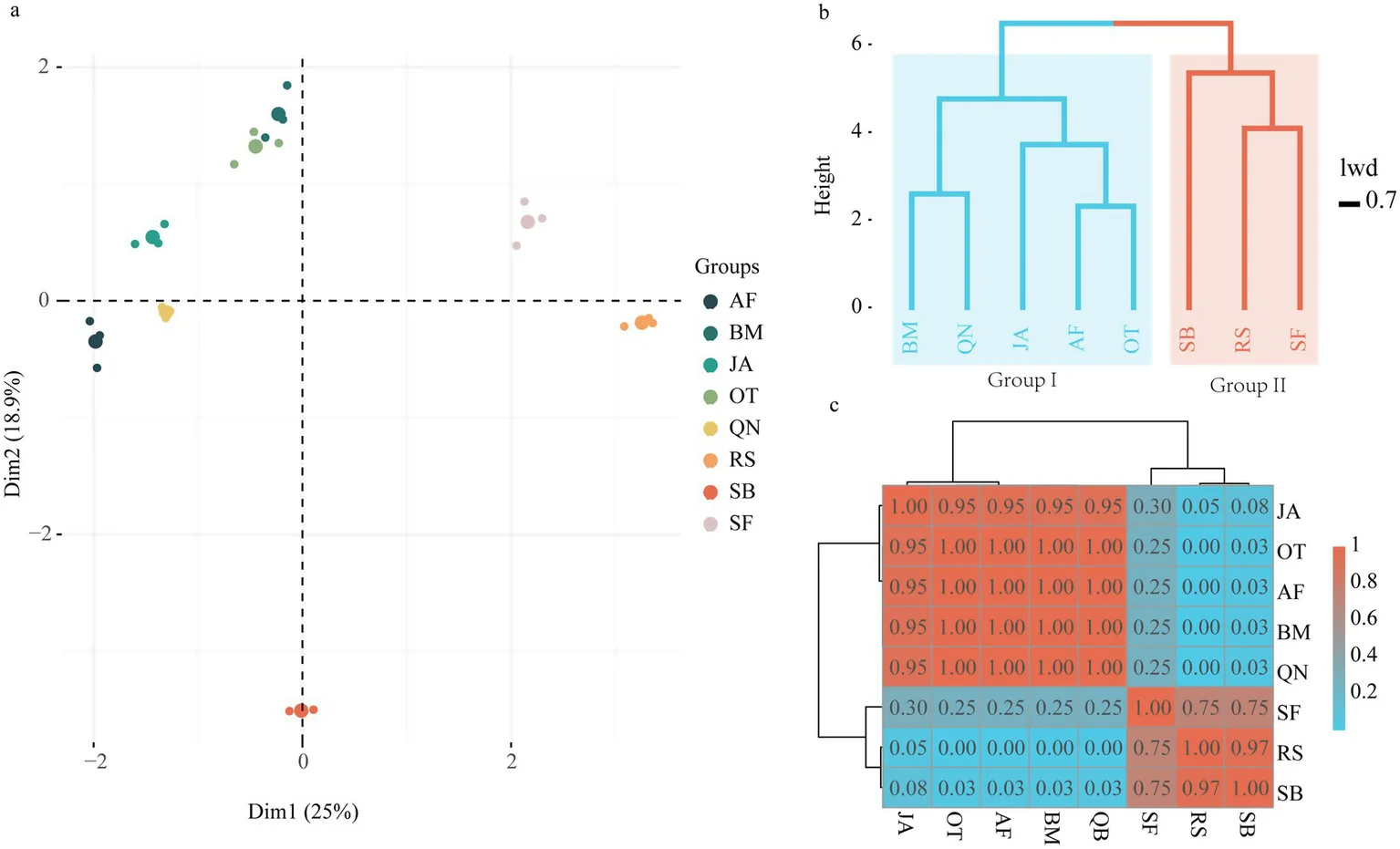
Integrated multiple factor analysis ordination and bootstrap-supported clustering of the eight crops. **(a)** Multiple factor analysis (MFA) ordination of the 24 biological samples. Dim 1 and Dim 2 explained 25.0 and 18.9% of the total variation, respectively, and colored points represent independent biological replicates of each crop. **(b)** Ward.D2 hierarchical clustering of crop centroids calculated from the retained MFA dimensions. The analysis separated the crops into Group I (BM, QN, JA, AF and OT) and Group II (SB, RS and SF). **(c)** Bootstrap co-clustering frequency matrix based on 1,000 within-crop resampling iterations. Values indicate the proportion of bootstrap iterations in which each pair of crops was assigned to the same cluster, with warmer colors indicating higher co-clustering stability and cooler colors indicating stronger separation.

The first four MFA dimensions retained for clustering explained 73.6% of the total variation, accounting for 25.0%, 18.9%, 16.3%, and 13.4%, respectively (Supplementary Figure S11). Contribution analysis showed complementary domain-specific patterns across these dimensions. Soil salinity and moisture contributed most strongly to Dim 1, whereas enzyme activities and plant nutrient–ion traits were the major contributors to Dim 2. Dim 3 was dominated by microbial diversity and community structure together with soil nutrient characteristics, while network and inferred functional characteristics contributed most strongly to Dim 4. At the individual-variable level, DHA and urease showed the highest weighted contributions across the retained MFA dimensions (8.6% and 7.4%, respectively), followed by soil total salts, moisture content, water-soluble Na^+^ and several nutrient variables. These results indicate that the MFA separation reflected complementary contributions from multiple soil, plant and microbial domains rather than variation in a single trait set.

## Discussion

### Crop identity reconstructed the same saline-alkaline soil into distinct rhizosphere ecological niches

Crop identity was associated with marked differentiation in rhizosphere soil, plant ion and microbial characteristics under a common saline-alkaline field background. This is consistent with current plant microbiome frameworks in which host traits, soil conditions and microbial ecological processes jointly shape rhizosphere communities (Compant et al., 2025; Russ et al., 2023). Crop-specific differences in nutrient uptake, rhizodeposition, ion allocation and microbial recruitment can generate contrasting rhizosphere microenvironments even under shared field conditions (Faist et al., 2023; Zhao et al., 2025). Together, these differences show that crop identity was associated with distinct rhizosphere environments under a shared field background.

Sampling was aligned with crop phenology to improve biological comparability among species by minimizing developmental-stage heterogeneity, and the two sampling dates were confined to a 16-d interval within the same seasonal window, with no irrigation or other major field-management operations conducted between them. The common maize preceding crop should also be considered when interpreting these differences. Maize may have left residual effects on soil nutrient availability and microbial community composition, but because all plots shared the same preceding-crop history and subsequent management, this legacy represented a common background rather than a crop-specific treatment factor. Nevertheless, carry-over effects cannot be completely excluded, and multi-year rotation experiments will be needed to further separate current-crop effects from longer-term crop legacy effects.

### Crop-specific Na^+^–K^+^ allocation reflects contrasting tissue ion partitioning

The contrasting Na^+^–K^+^ allocation patterns indicate that crops differed not only in tissue ion concentrations but also in the distribution of Na^+^ and K^+^ between roots and aboveground tissues. Quinoa combined high K^+^ concentrations with low Na^+^/K^+^ ratios, a characteristic consistent with maintenance of K-dependent metabolic and osmotic functions under saline conditions. Sunflower showed a different allocation pattern, with high root Na^+^, restricted Na^+^ translocation and strong K^+^ translocation to aboveground tissues, indicating greater root-associated Na^+^ distribution while maintaining K^+^ supply to shoots. In contrast, sugar beet showed high aboveground Na^+^ and the highest Na^+^ translocation factor, indicating preferential Na^+^ distribution to aboveground tissues.

These patterns describe crop-specific tissue ion partitioning rather than whole-plant salt tolerance, highlighting substantial variation in Na^+^–K^+^ distribution among crops under saline-alkaline field conditions.

### Rhizosphere conditions were associated with microbial community differentiation

Microbial community differentiation was closely associated with the crop-specific rhizosphere environments established under the common saline-alkaline field background. Previous studies have shown that plant-associated microbiomes are shaped jointly by host identity, resource availability and local environmental filtering, rather than by soil background alone (Seabloom et al., 2023; Sessitsch et al., 2023). In the present study, TN, OC, TP and available N showed coordinated variation, while water-soluble Na^+^, total salts and soil moisture were also closely associated with one another. Together with the RDA and Mantel results, these patterns indicate that microbial community differentiation was associated with variation in both nutrient status and salinity–moisture conditions. The contrasting positions of crops along these gradients indicate that crop identity altered the physicochemical context in which microbial communities assembled. This interpretation is consistent with the concept that rhizosphere microorganisms respond to crop-driven changes in nutrient supply, rhizodeposition and local stress conditions, thereby generating host-associated microbial assemblages even within the same field soil (Chen et al., 2024; Faist et al., 2023).

The bacterial and fungal communities responded to this environmental differentiation in distinct ways. Bacterial communities retained a relatively large shared OTU core, whereas fungal communities showed stronger crop-associated compositional differentiation, suggesting that the two microbial kingdoms differed in their sensitivity to crop-specific rhizosphere conditions. Alpha-diversity patterns supported this distinction: bacterial richness, Shannon diversity and phylogenetic diversity showed clear pairwise differences among several crops, whereas fungal richness and Faith’s PD showed significant overall crop effects but weaker pairwise separation. More importantly, crop-specific biomarkers and environment-responsive OTUs occurred within the dominant bacterial and fungal lineages, indicating that crop identity mainly altered the relative representation of particular microbial groups rather than completely replacing the resident community. This pattern agrees with current plant microbiome frameworks in which roots selectively recruit or enrich subsets of a common soil microbial pool through resource inputs and local physicochemical gradients (Chen et al., 2024; Sessitsch et al., 2023).

These compositional differences were accompanied by changes in microbial co-occurrence organization. Sunflower and rapeseed supported more densely connected networks, whereas Jerusalem artichoke and oat showed relatively high network complexity, indicating that crop identity influenced not only which taxa were present but also how microbial associations were organized. Similar studies have shown that microbial network structure can shift markedly under environmental disturbance and host-driven changes in resource conditions, with potential consequences for community stability and ecological organization (Cairns et al., 2025; Knight et al., 2024). The PICRUSt2 analysis provided an additional, exploratory layer by suggesting crop-associated differences in inferred pathways related to carbon metabolism, amino acid metabolism, chemotaxis, antioxidant defence and DNA repair. Comparable crop-dependent functional differentiation has also been reported in crop rhizobacteriomes under field conditions (Abdelrahman et al., 2025). Although these predicted functions require validation using direct functional approaches (Douglas et al., 2020), their consistency with the observed taxonomic, environmental and network patterns supports the broader interpretation that different crops generated distinct microbial ecological organizations under the same saline-alkaline soil background.

### Integrated trait differentiation underlies the two MFA groups

The MFA contribution structure provides an integrative explanation for the separation of the eight crops into two multivariate groups. Importantly, no single trait domain dominated all major MFA dimensions. Instead, the retained dimensions captured complementary components of crop-associated variation: rhizosphere salinity and moisture contributed most strongly to Dim 1, enzyme activity and plant nutrient–ion traits to Dim 2, soil nutrient status and microbial community structure to Dim 3, and microbial network and inferred functional characteristics to Dim 4. This multidimensional pattern indicates that crop differentiation under the same saline-alkaline field background cannot be attributed to a single salinity gradient or plant ion trait. Rather, the observed grouping reflected coordinated variation in rhizosphere physicochemical conditions, biochemical activity, plant ion allocation and microbial organization.

The high weighted contributions of DHA and urease, together with those of total salts, soil moisture, water-soluble Na^+^ and nutrient variables, further highlight the importance of the rhizosphere biochemical and physicochemical environment in distinguishing crop-associated profiles. At the same time, the substantial contributions of plant nutrient–ion traits and microbial diversity, community structure and network characteristics across different MFA dimensions show that these environmental differences were embedded within a broader soil–plant–microbe response. This interpretation is consistent with current frameworks in which crop-associated rhizosphere functioning emerges from interactions among host traits, soil conditions and microbial communities rather than from isolated environmental variables (Russ et al., 2023; Zhao et al., 2025).

Accordingly, the two MFA groups are more appropriately interpreted as broad multivariate crop-associated profiles than as discrete physiological strategies. Group I combined generally lower rhizosphere salinity with relatively stronger nutrient- and enzyme-related characteristics, whereas Group II combined higher salinity with greater contributions from Na^+^ allocation traits. The value of this classification therefore lies in identifying recurrent combinations of rhizosphere, plant and microbial traits that distinguish crops under a common field background. Linking these multivariate profiles with biomass, yield and longer-term field performance in future experiments will further clarify their relevance to crop selection and saline-alkaline land management.

## Conclusion

This study demonstrates that eight crops grown under a common saline-alkaline field background developed distinct multivariate soil, plant and microbial profiles. Crop identity was associated with coordinated differences in rhizosphere nutrient status, salinity and moisture, enzyme activities, plant Na^+^ and K^+^ allocation, microbial community composition and co-occurrence organization. Integrated multiple factor analysis separated the crops into two major multivariate profiles, one characterized primarily by stronger nutrient and enzyme traits and the other by higher rhizosphere salinity and greater Na^+^ allocation. At the crop level, quinoa, sunflower and sugar beet exhibited contrasting ion allocation characteristics, reflecting different patterns of K^+^ maintenance and Na^+^ distribution between roots and aboveground tissues. Together, these results show that crop-associated differentiation in saline-alkaline farmland emerges from coordinated variation across soil, plant and microbial domains rather than from salinity alone (Figure 6). This integrated framework provides a basis for identifying informative rhizosphere and plant traits for future crop evaluation and saline-alkaline soil management.

**Figure 6:**
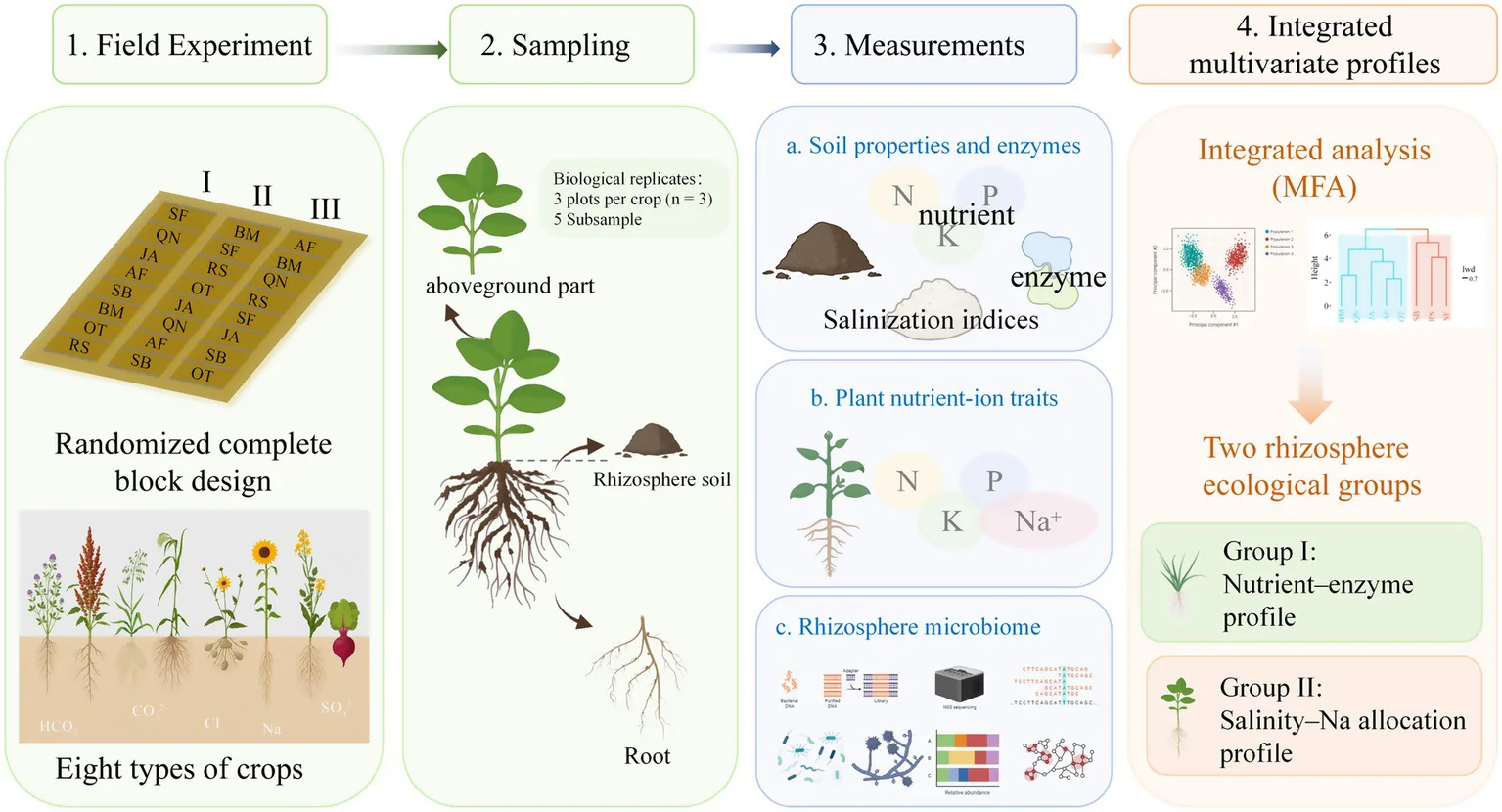
Overview of the experimental design and integrated analytical framework.

## Data Availability

The raw sequence data generated in this study have been deposited in the Genome Sequence Archive (GSA) of the National Genomics Data Center, China National Center for Bioinformation/Beijing Institute of Genomics, Chinese Academy of Sciences, under accession CRA034172. The data are publicly available at: https://ngdc.cncb.ac.cn/gsa.
