# Supplementary material for: Crop-associated differentiation of rhizosphere microbial communities and soil functional traits in saline-alkaline farmland
Source: Front Microbiol. 2026 Oct 15;17:1952914. doi: 10.3389/fmicb.2026.1952914 (PMC13716761; doi:10.3389/fmicb.2026.1952914)
Supplement: Supplementary file 1 [file Supplementary_File_1.docx]

**Fig. S1 Additional rhizosphere soil physicochemical properties across eight crops.**
Panels show (a) OC (g kg⁻¹), (b) TP (g kg⁻¹), (c) TK (g kg⁻¹), and (d) MC (%). Boxplots show the median and interquartile range, and individual points represent three biological replicates.

**Fig. S2 Shoot and root nutrient and Na⁺ concentrations across eight crops.**
Panels show (a) shoot total nitrogen (S-TN), (b) shoot total phosphorus (S-TP), (c) shoot total potassium (S-TK), (d) shoot Na⁺ concentration (S-Na⁺), (e) root total nitrogen (R-TN), (f) root total phosphorus (R-TP), (g) root total potassium (R-TK), and (h) root Na⁺ concentration (R-Na⁺), all expressed as g kg⁻¹. Boxplots show the median and interquartile range, and individual points represent three biological replicates. S- denotes aboveground tissues and R- denotes roots.

**Fig. S3 Shannon–Wiener rarefaction curves of rhizosphere bacterial and fungal communities.**
Rarefaction curves are shown for bacterial (a) and fungal (b) communities across the eight crops. The x-axis represents the number of sequencing reads sampled and the y-axis represents the Shannon diversity index. Each curve represents one biological replicate, with three biological replicates per crop.

**Fig. S4 Bacterial and fungal alpha diversity across the eight crops.**
Panels show bacterial Chao1 richness (a), Shannon diversity (b), and Faith’s phylogenetic diversity (PD) (c), and fungal Chao1 richness (d), Shannon diversity (e), and Faith’s PD (f). Boxplots show medians and interquartile ranges, and individual points represent three biological replicates. Overall crop effects were assessed using Kruskal–Wallis tests, with global P values adjusted across the six alpha-diversity indices using the Benjamini–Hochberg false discovery rate (FDR) procedure. For indices with significant overall crop effects, pairwise comparisons were conducted using Dunn’s post hoc test with Benjamini–Hochberg adjustment within each index. Different lowercase letters indicate significant pairwise differences at adjusted P < 0.05; crops sharing the same letter are not significantly different.

**Fig. S5 Beta-diversity patterns and shared OTUs of bacterial and fungal communities across the eight crops.**
Principal coordinate analysis (PCoA) based on Bray–Curtis dissimilarities is shown for bacterial (a) and fungal (b) communities. PCoA1 and PCoA2 explained 25.89% and 16.13% of bacterial community variation and 31.68% and 22.75% of fungal community variation, respectively. Permutational multivariate analysis of variance (PERMANOVA) indicated significant effects of crop identity on bacterial (R² = 0.781, P = 0.001) and fungal (R² = 0.835, P = 0.001) community composition. UpSet plots show shared and unique bacterial (c) and fungal (d) OTUs among crop groups. Vertical bars indicate intersection sizes, horizontal bars indicate total OTU set sizes for each crop, and the connected-dot matrix identifies the crop combinations represented by each intersection.

**Fig. S6 Taxonomic composition of rhizosphere bacterial and fungal communities at different taxonomic levels.**
Relative abundance profiles are shown for bacterial and fungal communities at the class (a, b), order (c, d), family (e, f), and genus (g, h) levels, respectively. Stacked bars represent the relative abundance of dominant taxonomic groups in each crop rhizosphere, with low-abundance or unresolved taxa grouped as appropriate in the corresponding legends.

**Fig. S7 Crop-specific bacterial–fungal co-occurrence subnetworks across the eight crop rhizospheres.**
Crop-specific subnetworks were extracted from the reference bacterial–fungal co-occurrence network by retaining OTUs detected in each crop and their corresponding associations in the reference network. Nodes represent microbial OTUs and are colored by microbial kingdom, with bacteria shown in blue and fungi in orange-red. Edges represent retained Spearman associations satisfying |r| ≥ 0.8 and Benjamini–Hochberg-adjusted P ≤ 0.05. Network edges represent statistical associations and should not be interpreted as direct biological interactions.

**Fig. S8 Modular organization of crop-specific rhizosphere microbial co-occurrence networks.**
The five largest modules identified using the fast-greedy modularity optimization algorithm are highlighted for each crop-specific subnetwork. Nodes and within-module connections are colored according to Module 1–Module 5, whereas nodes outside the five largest modules are shown in gray. The figure illustrates differences in modular organization among the eight rhizosphere microbial networks. Edges represent statistical co-occurrence associations rather than direct biological interactions.

**Fig. S9 PICRUSt2-inferred bacterial functional profiles across the eight crop rhizospheres.**
(a) Principal component analysis (PCA) of inferred bacterial functional profiles, shown as pairwise projections of the first three principal components. PC1, PC2 and PC3 explained 38.8%, 20.0% and 16.0% of the total variation, respectively. Symbols and colors represent samples from the eight crop groups. (b) Heatmap showing standardized predicted abundances of selected Kyoto Encyclopedia of Genes and Genomes (KEGG) pathways across crop groups. Red and blue indicate relatively higher and lower predicted pathway abundance, respectively. These profiles represent taxonomically inferred functional potential from 16S rRNA amplicon data and should not be interpreted as direct measurements of pathway activity.

**Fig. S10 Standardized representative traits associated with the two MFA groups.**
The heatmap shows standardized z-scores of representative rhizosphere and plant traits for each crop. Traits are organized into three domains: rhizosphere salinity, alkalinity and moisture (pH, WS-Na, TS and MC); rhizosphere nutrient status and enzyme activity (TN, OC, AN, Urease and DHA); and plant Na⁺–K⁺ allocation (shoot K⁺, shoot Na⁺, shoot Na⁺/K⁺, root K⁺, root Na⁺, root Na⁺/K⁺, Na⁺ translocation factor and K⁺ translocation factor). Rows correspond to individual crops, with side colors indicating MFA Group I and Group II. The two bottom rows show the mean standardized values for each MFA group. Warmer colors indicate above-average values and cooler colors indicate below-average values.

**Fig. S11 Contributions of variable blocks and individual variables to the retained MFA dimensions.**
(a) Contributions of the six MFA variable blocks to Dim 1–Dim 4, which explained 25.0%, 18.9%, 16.3% and 13.4% of the total variation, respectively. Blocks comprise soil nutrients, soil salinity and moisture, enzyme activities, plant nutrient and ion traits, microbial diversity and community structure, and network and inferred-functional characteristics. The dashed horizontal line represents the expected average contribution under equal block contributions (100/6 = 16.7%). (b) Variables with the highest weighted contributions across the four retained MFA dimensions. Bar colors indicate the corresponding variable block, and values at the ends of bars show weighted contribution percentages. The dashed vertical line represents the expected average contribution under equal contributions of the 44 variables (100/44 ≈ 2.27%).
